# Neurological symptoms and physical exam findings 6–11 months post-COVID-19: a cohort study

**DOI:** 10.1038/s41598-025-33779-w

**Published:** 2026-01-02

**Authors:** Bruno Fukelmann Guedes, Ana Paula Ritto, Andre Macedo Serafim Silva, Antonio Edvan Camelo Filho, Cristiane de Araújo Martins Moreno, Marcos Vinícius Oliveira Marques, Mariana Ribeiro Marcondes da Silveira, Raphael de Luca eTuma, José Pedro Soares Baima, Sâmia Yasin Wayhs, Rodrigo de Holanda Mendonça, Cristiana Borges Pereira, Bruno Diógenes Iepsen, Barbara Leite Costa, Emanuelle Roberta da Silva Aquino, Julia Chartouni Rodrigues, Geraldo Busatto Filho, Edmar Zanoteli, Suely Kazue Nagahashi Marie, Ricardo Nitrini, Luiz Henrique Martins Castro

**Affiliations:** 1 Department of Neurology, Faculdade de Medicina da Universidade de São Paulo, São Paulo, SP Brazil; 2 Hospital das Clínicas da Faculdade de Medicina da Universidade de São Paulo, São Paulo, Brazil; 3 Department of Psychiatry, Faculdade de Medicina da Universidade de São Paulo, São Paulo, SP Brazil; 4 Division of Neurology, Department of Clinical Medicine, Universidade Federal do Ceará, Fortaleza, CE Brazil; 5 Saint Louis University Hospital, St Louis, USA

**Keywords:** Post-Acute COVID-19 syndrome, Neurologic manifestations, Peripheral nervous system diseases, Neuromuscular diseases, Neurological manifestations, Neurology, Cerebrovascular disorders, Epilepsy, Peripheral neuropathies, SARS-CoV-2

## Abstract

Somatic neurological complications are an underexplored component of post-acute sequelae of SARS-CoV-2 infection. We evaluated long-term somatic neurological symptoms, examination findings, and suspected diagnoses among adults hospitalized for COVID-19. A total of 708 survivors were assessed 6–11 months after discharge using a structured three-step neurological evaluation based on the World Health Organization *Protocol for Epidemiologic Studies of Neurologic Disorders*: an adapted symptom questionnaire, a brief standardized neurological examination, and a full neurological consult. Muscle weakness, gait impairment, and paresthesias remained the most frequent symptoms at follow-up. Longer hospital stay predicted all three symptoms, and diabetes was associated with both gait impairment and paresthesias. Common examination findings included monoparesis, hemiparesis, and gradient-pattern sensory loss. Clinically suspected diagnoses included neuromuscular disease in 26%, cerebrovascular disease in 10%, and epilepsy in only 2.7% of cases. Logistic regression linked neuromuscular disease to paresthesias and tandem-walk failure, cerebrovascular disease to facial paralysis, and epilepsy to loss-of-contact episodes. A simplified screening model derived through best-subsets selection identified five informative items from the WHO protocol: paresthesias, facial paralysis, tandem walk, index–nose test, and loss-of-contact episodes. These findings indicate that somatic neurological abnormalities remain frequent months after severe COVID-19 and that a brief bedside subset may support efficient post-COVID neurological triage.

**Supplementary Information:**

The online version contains supplementary material available at 10.1038/s41598-025-33779-w.

## Introduction

Neurological complications of acute coronavirus disease 2019 (COVID-19) were widely documented early in the pandemic^1–7^. Approximately 36% of hospitalized COVID-19 patients in Wuhan exhibited neurological manifestations^2^. As the pandemic progressed, attention shifted toward the long-term consequences of SARS-CoV-2 infection. Many patients continued to experience persistent symptoms affecting multiple organ systems after the acute phase, a condition now referred to as *long COVID* or *post-acute sequelae of SARS-CoV-2 infection (PASC)*^8,9^. Notably, neurological and neuropsychiatric symptoms are among the most frequent and debilitating features of long COVID. In a large electronic health record cohort of 236,379 COVID-19 survivors, the estimated 6-month incidence of any neurological or psychiatric diagnosis was 33.6%^10^. Likewise, fatigue and muscle weakness have been observed in over half of survivors at 6-month follow-up^11^. These findings underscore the substantial long-term neurological burden faced by COVID-19 survivors and the need for dedicated research in this area.

While cognitive and psychiatric sequelae of COVID-19 have been extensively studied^9,12,13^ somatic neurological complications (such as neuromuscular and other peripheral nervous system disorders) remain comparatively under-characterized.

There is increasing evidence that COVID-19 can impact both the central and peripheral nervous systems^14^. Multiple case reports and series have described peripheral nervous system involvement – for example, Guillain–Barré syndrome and various cranial neuropathies – in the post-COVID context^14^. However, detailed in-person neurological examination data from large cohorts of COVID-19 survivors are scarce. A few prospective studies have evaluated long-term neurological outcomes with specialist examinations^15–19^, but their findings have varied widely. Important limitations of prior studies include relatively small sample sizes (often < 200 patients)^16–19^, cohorts dominated by mild/non-hospitalized^17^, in-person neurological evaluation in only a fraction of cases^18,19^ and absence of a standardized neurological exam protocol^17–19^. Such heterogeneity complicates comparisons and may underestimate the prevalence of subtle neurological deficits in more severely ill survivors.

We aimed to (1) characterize the burden and pattern of somatic neurological symptoms and examination abnormalities at a medium-term post-discharge visit among adults hospitalized for moderate-to-severe COVID-19 (planned ≈ 6 months; observed window 6–11 months); (2) identify clinical correlates of these symptoms and signs, focusing on baseline comorbidities and indicators of acute illness severity; (3) classify clinically suspected neurological diagnoses (neuromuscular, cerebrovascular, epilepsy) based on a structured neurologist assessment; and (4) evaluate the informativeness of a brief epidemiological screening tool set, examining whether a concise subset of bedside items can flag patients at risk for major somatic neurological diagnoses.

## Methods

### Study population

This study was conducted at Hospital das Clínicas da Faculdade de Medicina da Universidade de São Paulo (HC-FMUSP), a tertiary university hospital, that was partially repurposed as a COVID-only facility. During most of the year of 2020, the hospital’s Instituto Central became a COVID-dedicated unit with 900 beds (including three hundred intensive care unit beds).

Adult patients hospitalized at HC-FMUSP for at least 24 h with moderate-to-severe forms of COVID-19 between March 30th and August 30th, 2020, were considered eligible for this study.

We excluded patients with nosocomial COVID-19, severe dementia, or those living in long-term care facilities; as well as individuals who died, were unable to attend in-person visits 6 months after discharge due to reduced mobility, had suspected reinfection, refused participation, or did not complete the neurological evaluation.

After signing informed consent, the study subjects underwent extensive multidisciplinary evaluation at a dedicated research outpatient clinic between 6 and 11 months after hospital discharge.

The present study was part of a larger multidisciplinary project that is described in greater detail elsewhere^8^. This study has been approved by the Ethics Committee at HCFMUSP (CAPPesqHC, CAEE: 33787520.4.0000.0068) and registered in the Brazilian Registry of Clinical Trials (ReBEC) under the registration number 4.270.242 (RBR-8z7v5wc). It is reported according to the Strengthening the Reporting of Observational Studies in Epidemiology (STROBE) Statement^20^. All research procedures involving human participants were performed in accordance with relevant guidelines and regulations, including the Declaration of Helsinki. Cognitive and neuropsychiatric findings from the same cohort have been previously reported^12,21,22^ .

### Clinical evaluation

#### Epidemiological and acute COVID-19 data

Information on demographic variables, baseline comorbidities, and acute COVID-19–related clinical data were obtained from hospital electronic charts and databases. Baseline characteristics included age, sex, diabetes, hypertension, chronic kidney disease, previous stroke, previous myocardial infarction, and body-mass index. Acute COVID-19 variables included total length of hospital stay, admission to the intensive care unit (ICU) and ICU length of stay, and need for orotracheal intubation.

#### Adapted WHO protocol

Although the cohort was recruited prospectively for in-person evaluation, all data were collected at a single follow-up visit between 6 and 11 months after hospital discharge. During this visit, participants underwent a structured three-step neurological evaluation performed by board-certified neurologists, neurosurgeons, or pediatric neurologists. Data were captured and stored in real time using web-based case report forms developed on the Research Electronic Data Capture (REDCap) system hosted at HCFMUSP (https://www.redcapbrasil.com.br/*).*

##### Steps 1–2 (adapted WHO screening protocol)

The first two steps together constituted the adapted screening protocol derived from the World Health Organization tool for neuroepidemiologic surveys in low- and lower-middle-income countries^23^.

Step 1 was an interviewer-administered questionnaire, freely translated into Brazilian Portuguese and adapted to capture potentially COVID-related symptoms across three recall anchors. For continuous/longitudinal symptoms, participants were asked whether they had each symptom (1) before COVID-19, (2) at hospital discharge, and (3) at the follow-up visit. The follow-up question (“Do you still have this symptom now?”) was displayed only if the participant had reported the symptom at discharge, following the skip-logic structure of the electronic case-report form. Consequently, the follow-up responses represent symptom persistence among discharge-positive participants, not overall prevalence or new-onset symptoms. All subsequent analyses of follow-up data were therefore conditional on having had the symptom at discharge. Paroxysmal symptoms—such as headache and loss of consciousness—were recorded as having occurred at any time before COVID-19 and at any time after COVID-19. Their episodic nature does not permit defining a point-prevalence at discharge, so skip-logic was not used.

Step 2 was a brief neurological examination, unchanged from the original WHO protocol, comprising seven tasks: outstretched-arms maneuver, matchstick pick-up, discrimination of smooth versus rugged cloths, index–nose testing, two-meter tandem walk, feet-together balance, and feet-together with eyes closed (Romberg’s) test.

The complete WHO screening protocol (Steps 1 + 2 in combination) has demonstrated high diagnostic performance in population neuroepidemiology (e.g., the Sicilian Neuro-Epidemiologic Study: sensitivity 96%, specificity 86%)^24^, with validations and adaptations reported in Africa^25–27^, India^28–30^, and Latin America^31,32^ .

##### Step 3 (structured neurological consult)

All participants subsequently underwent a brief clinical history and detailed neurological examination assessing motor strength, deep-tendon reflexes, pyramidal signs, pinprick and vibration sensation, cranial-nerve function, movement disorders, meningeal signs, balance, and coordination. At the end of Step 3, the physician recorded suspected neurological diagnoses as short free-text notes. The full protocol, including questionnaires and examination methods, is provided as Supplemental File 1.

To reduce inter-rater variability, all raters met individually with the principal investigator (BFG) for protocol clarification and training. The PI directly supervised the first five cases evaluated by each physician to ensure alignment before independent assessments. The structured data-collection form was included in the original protocol (Busatto et al.^8^, pages 24–31, and Supplementary File 1). All examination findings and diagnoses were reviewed by the principal investigator for consistency and reclassified into specific post-hoc diagnostic categories.

### Classification of clinical diagnoses

Based on the diagnostic impressions documented in the records, each case was assigned to one of four major categories: cerebrovascular disease, neuromuscular disorders, epilepsy, or other conditions. Classification was performed using predefined keyword sets; for example, the label “neuromuscular disorder” was applied when terms such as *neuropathy* or *small-fiber* appeared in the text. A full list of keywords is provided in Supplementary File 2. Diagnoses reflected clinical hypotheses only, as ancillary testing was not available in the study.

#### Validation of clinical diagnoses

To evaluate the internal consistency of research-derived clinical diagnoses for neuromuscular disorders, epilepsy, and cerebrovascular disease, we compared them with available ancillary test results (EMG, EEG, or brain imaging) in a convenience subsample. These tests were obtained at the discretion of treating clinicians and were not mandated by the study protocol; consequently, patients with normal findings are under-represented. Accordingly, these comparisons are presented descriptively as internal consistency checks, rather than formal validation analyses, since inferential statistics (e.g., Cohen’s κ, Fisher’s exact test) would be biased and uninformative under such opportunistic sampling.

### Statistical analysis

#### Prevalence and within-subject changes in symptoms

Each non-paroxysmal symptom was assessed at three time points: before COVID-19 (baseline), at hospital discharge, and at follow-up. Follow-up questions were displayed only to participants who had reported the symptom at discharge; therefore, follow-up values represent persistent symptoms. Our prespecified hypothesis was that symptoms would remain more prevalent at follow-up than before COVID-19.

To assess within-individual change for each symptom, we compared baseline prevalence with follow-up persistent prevalence using the one-sided exact McNemar test (alternative = “greater”). This directional approach reflects the expectation of increased symptom burden in the post-COVID period. P-values were adjusted for multiple comparisons using the Benjamini–Hochberg false discovery rate (FDR) method.

##### Logistic regression analysis of symptom persistence: associations with baseline and acute COVID-19 characteristics

Following the initial analysis of symptom prevalence over time based on the adapted WHO protocol, logistic regression models were employed to evaluate the associations between potential baseline and acute COVID-19 characteristics and the symptoms identified as significantly more prevalent on follow-up from McNemar’s tests. The baseline and acute COVID-19 characteristics included age, sex, diabetes, hypertension, previous stroke, length of hospital stay, intensive care admission, and intubation. The models were fitted separately for each symptom to estimate the odds ratios (OR) and 95% confidence intervals (CI) for each predictor.

#### Major potential diagnoses and the WHO protocol

##### Global logistic regression models

Initial analyses utilized logistic regression models to evaluate the association between potential predictors and the three major diagnoses (epilepsy, neuromuscular diseases, and cerebrovascular diseases), which were determined through structured neurological consultations, and classified post-hoc based on recorded clinical hypotheses. All elements from the WHO protocol were included as potential predictors in the global models. The models were fitted separately for each diagnosis to estimate the odds ratios (OR) and 95% confidence intervals (CI) for each predictor. Given the potential for separation, quasi-separation, and small-sample bias, Firth’s correction was applied^33,34^.

##### Best subsets selection method

To identify the optimal subset of predictors for each neurological condition—epilepsy, neuromuscular diseases, and cerebrovascular diseases—we applied an exhaustive best subsets selection using logistic regression models with Firth’s correction. This approach systematically evaluates all possible predictor combinations to determine the best-fitting model for each dependent variable. We selected the model with the lowest Bayesian Information Criterion (BIC) as optimal, as BIC penalizes model complexity, thereby favoring more parsimonious models^35^. This process allowed us to identify the most informative predictors while controlling for small-sample bias and separation issues commonly encountered with rare outcomes. For the final selected models, we presented odds ratios for the outcomes. Because coefficients were refitted after variable selection, parameter estimates and p-values may be biased toward significance.

### Software and computation resources

All statistical analyses were conducted using R (version 4.4.1) and the following packages: descTools, stats, exact2 × 2, rcompanion, logistf, brglm2. The best subsets selection method was performed using a custom script with a high-memory, high-core count cloud computing instance, requiring several minutes of runtime. The analysis is fully reproducible by cloning the GitHub repository (https://github.com/GuedesBF/Neurological-Symptoms-and-Exam-Findings-6-11-Months-Post-COVID-19.git) and rendering the Quarto document within RStudio. The rendered Quarto file is provided as Supplementary File 3.

## Results

Between March 30 and August 31, 2020, 3,009 patients were hospitalized with COVID-19, 1,957 survived hospitalization, and 708 cases were included in the final analysis (Supplementary Fig. 1, Study Flow Diagram). The sample was largely composed of severe cases (57.5% were admitted to the ICU and 39.3% were intubated). Follow-up visits occurred a median of 212 days [IQR 200–249] after discharge. Baseline characteristics were similar between ICU- and non–ICU-admitted patients (Table 1).

**Table 1:** Baseline and acute COVID-19 characteristics.

	ICU non-admitted N = 301^*1*^	ICU-admitted N = 407^*1*^
Baseline characteristics		
Age	56 [44–66]	57 [44–65]
Sex—female	143 (48%)	192 (47%)
Diabetes	97 (32%)	151 (37%)
Hypertension	164 (55%)	237 (58%)
Chronic kidney disease	40 (14%)	25 (6.3%)
Previous cerebrovascular disease	11 (3.7%)	20 (4.9%)
Previous myocardial infarction	25 (8.4%)	38 (9.6%)
Body mass index	29 [26–34]	28 [24–34]
Acute COVID characteristics		
Length of hospital stay	6 [4–9]	20 [13–33]
Intensive care—length of stay	-	10 [6–18]
Intubation	1 (0.3%)	275 (68%)

^*1*^ median [Q1–Q3]; n (%)

Values are median [Q1–Q3] for continuous variables and counts (n) with percentages for categorical variables.

### WHO protocol findings

Four symptoms remained significantly more common at follow-up compared with their pre–COVID-19 baseline levels. Muscle weakness (7.6% → 15.5%), gait impairment (9.9% → 15.3%), paresthesias (11.7% → 22.0%), and speech impairment (5.8% → 10.7%) all showed persistent excess, with FDR-adjusted one-tailed McNemar p-values < 0.001 (Table 2). Other symptoms showed no meaningful paired change.

**Table 2:** Longitudinal comparison of symptom prevalence.

	Before COVID	At discharge	Follow-up	McNemar’s *
Loss of consciousness	7.2%	–	3.2%	> 0.999
Loss of contact	6.5%	–	3.8%	> 0.999
Tremors or spasms	8.1%	14.1%	9.7%	0.195
Speech impairment	5.8%	–	10.7%	< 0.001
Facial paralysis	3.0%	4.5%	3.4%	0.607
Facial pain	4.8%	1.1%	1.0%	> 0.999
Muscle weakness	7.6%	28.1%	15.5%	< 0.001
Gait impairment	9.9%	33.6%	15.3%	0.001
Paresthesias	11.7%	26.4%	22.0%	< 0.001
Headache	41.0%	–	40.3%	0.975

- : unavailable / not assessed.

* p values from one-tailed exact McNemar’s tests. Null hypothesis: baseline(before Covid) is not lower than follow-up (symptoms present at discharge and persistent at follow-up). Values are adjusted with Benjamini-Hochberg (BH) procedure.

Physical examination items of the WHO protocol were less informative, with most abnormalities occurring in < 10% of patients, except for tandem walk failure (18%) (Table 3). As expected, some measures were interrelated (e.g., balance and Romberg tests), as illustrated in Supplementary Fig. 2.

**Table 3:** Physical examination findings (WHO protocol – Step 2).

WHO protocol—step 2	
Characteristic	N = 708
Arms extended	
Bilateral deficit	6 (0.9%)
Normal	678 (96%)
Unilateral deficit	20 (2.8%)
Missing/not tested	4
Match stick pick-up	
Bilateral deficit	11 (1.6%)
Normal	658 (95%)
Unilateral deficit	22 (3.2%)
Missing/not tested	17
Cloth discrimination test	
Bilateral deficit	8 (1.1%)
Normal	680 (97%)
Unilateral deficit	13 (1.9%)
Missing/not tested	7
Index-nose test	
Bilateral deficit	10 (1.4%)
Normal	670 (96%)
Unilateral deficit	17 (2.4%)
Missing/not tested	11
Tandem walk test	
Complete deficit/incapable	91 (13%)
Normal	567 (82%)
Partial deficit/difficulty	33 (4.8%)
Missing/not tested	17
Balance test	
Complete deficit/incapable	23 (3.3%)
Normal	673 (97%)
Missing/not tested	12
Romberg’s test	
Complete deficit/incapable	11 (1.6%)
Could not stand	1 (0.1%)
Normal	657 (98%)
Missing/not tested	39

Findings from the standardized Step 2 physical examination of the WHO neurological screening protocol. Values are counts (n) and percentages of participants presenting each findings.

Modeling of predictors of persistent symptoms at follow-up are presented in Table 4. Longer hospital stay was consistently associated with higher odds of all studied symptoms (with odds increasing by roughly 2–3% per day of stay). Diabetes increased the odds of gait impairment and paresthesias.

**Table 4:** Logistic regression analysis of predictors for neurological symptoms.

Predictor	Speech impairment			Muscle weakness			Gait impairment			Paresthesias		
	OR	95% CI	p-value	OR	95% CI	p-value	OR	95% CI	p-value	OR	95% CI	p-value
Sex—male	0.86	0.53, 1.40	0.5	0.94	0.61, 1.44	0.8	1.06	0.68, 1.66	0.8	0.87	0.60, 1.25	0.4
Age	1	0.98, 1.02	0.8	0.99	0.97, 1.00	0.2	1	0.98, 1.02	0.8	0.99	0.98, 1.01	0.3
Diabetes	1.43	0.84, 2.40	0.2	1.56	0.98, 2.48	0.062	2.07	1.29, 3.33	**0.003**	1.55	1.04, 2.32	**0.031**
Hypertension	1.03	0.59, 1.84	> 0.9	0.93	0.56, 1.54	0.8	1.11	0.66, 1.90	0.7	1.14	0.74, 1.77	0.5
Previous stroke	1.77	0.61, 4.46	0.3	1.12	0.38, 2.85	0.8	3.95	1.67, 9.09	**0.001**	2.21	0.98, 4.84	0.05
Length of stay	1.02	1.01, 1.04	**0.003**	1.02	1.01, 1.04	** < 0.001**	1.03	1.01, 1.04	** < 0.001**	1.02	1.00, 1.03	**0.007**
Intensive Care admission	0.45	0.20, 0.93	**0.039**	1.5	0.80, 2.78	0.2	1.54	0.77, 3.05	0.2	0.92	0.52, 1.59	0.8
Intubation	1.09	0.50, 2.51	0.8	0.93	0.52, 1.69	0.8	1.32	0.72, 2.51	0.4	1.55	0.90, 2.71	0.12

Significant values are in [bold].

Odds ratios (OR), 95% confidence intervals (CI), and p-values from independent multivariable logistic regression models for each symptom. Each model was fitted independently and adjusted for all listed covariates. P-values are adjusted within but not across models.

### Comprehensive neurological examination and potential diagnoses

The semi-structured standard neurological examination showed a very low rate of cranial nerve abnormalities. Abnormalities were rare across all cranial nerve groups, with fewer than 5% of patients showing deficits in any single nerve group. Somatic examination was more informative. Relevant findings on motor testing included monoparesis (6.6%) and hemiparesis (6.2%). Sensory testing revealed gradient-type sensory loss in pinprick (13%) and vibration sense (16%). Pinprick sensation testing showed focal abnormalities in 9.5% of patients (Table 5).

**Table 5:** Structured physical examination (N = 708).

Somatic examination				Cranial nerves			
Motor		Vibration sense		II		VII	
Monoparesis	47 (6.6%)	Focal	17 (2.4%)	Unilateral vision loss	15 (2.1%)	Central dysfunction	21 (3.0%)
Hemiparesis	44 (6.2%)	Hemi hypoesthesia	12 (1.7%)	Hemianopia	6 (0.8%)	Peripheral dysfunction	9 (1.3%)
Paraparesis	13 (1.8%)	Gradient	111 (16%)	Bilateral amaurosis	1 (0.1%)	Other	4 (0.6%)
Quadriparesis	19 (2.7%)	Level	1 (0.1%)	Other	5 (0.7%)	Normal	674 (95%)
Other	14 (2.0%)	Other	11 (1.6%)	Normal	674 (95%)	Unclear/not tested	0 (0%)
Normal	571 (81%)	Normal	556 (79%)	Unclear/not tested	7 (1.0%)	VIII	
Unclear/not tested	0 (0%)	Unclear/not tested	0 (0%)	III-VI		Unilateral dysfunction	6 (0.8%)
Pinprick sensation		Involuntary movements		Normal	699 (99%)	Bilateral dysfunction	8 (1.1%)
Focal abnormalities	67 (9.5%)	Tremor	46 (6.5%)	Ophthalmoparesis	9 (1.3%)	Other	2 (0.3%)
Hemi hypoesthesia	29 (4.1%)	Parkinsonism	5 (0.7%)	Unclear/not tested	0 (0%)	Normal	692 (98%)
Gradient	90 (13%)	Myoclonus	3 (0.4%)	V		Unclear/not tested	0 (0%)
Level	6 (0.8%)	Other	0 (0%)	Unilateral dysfunction	15 (2.1%)	IX-XII	
Other	17 (2.4%)	Normal	654 (92%)	Other	3 (0.4%)	Bulbar dysfunction	6 (0.8%)
Normal	499 (70%)	Unclear/not tested	0 (0%)	Normal	686 (97%)	Normal	702 (99%)
Unclear/not tested	0 (0%)	Nuchal rigidity		Unclear/not tested	4 (0.6%)	Unclear/not tested	0 (0%)
Coordination		Absent	702 (99%)				
Cerebellar ataxia	7 (1.0%)	Present	3 (0.4%)				
Sensory ataxia	30 (4.2%)	Unclear/not tested	3 (0.4%)				
Normal	662 (94%)						
Unclear/not tested	9 (1.3%)						

Findings from the standardized neurological examination are summarized by domain. Values are counts (n) and percentages of participants presenting each finding.

Neuromuscular disease (the majority of cases, polyneuropathy), was the most common diagnosis, including 182 cases (25.7%), followed by 73 cases (10.3%) of cerebrovascular cases and 19 (2.7%) cases of epilepsy (Table 6).

**Table 6:** Suspected Somatic neurologic diagnoses at follow-up N = 708.

Condition	
Neuromuscular disease	
Any neuromuscular disease	182 (26%)
Polyneuropathy	126 (18%)
Mononeuropathies	
Any mononeuropathy	62 (8.8%)
Peroneal neuropathy	36 (5.1%)
Lateral femoral neuropathy	13 (1.8%)
Other lower limb neuropathies	5 (0.7%)
Median neuropathy	9 (1.3%)
Ulnar neuropathy	7 (1.0%)
Motor neuron disease	2 (0.3%)
Radiculopathy	5 (0.7%)
Cerebrovascular disease	
Cerebrovascular disease	73 (10%)
Epilepsy	
Epilepsy	19 (2.7%)
Other diagnoses	
Parkinsonism	6 (0.8%)
Tremor	17 (2.4%)

Diagnoses suspected based on the structured neurological consult on follow-up. Values are counts (n) and percentages of participants suspected for each condition.

### Consistency of clinical diagnoses with ancillary testing

The internal consistency of the clinically derived neurological diagnoses was evaluated using available ancillary studies obtained outside the study protocol. EMG reports were retrieved directly from the hospital’s electrophysiology database, whereas EEG and neuroimaging (CT or MRI) data were identified through manual review of 94 patient charts (convenience sample).

EMG studies were available for 23 patients (3.2% of the cohort), of which only two were normal. Electrophysiological results were broadly consistent with the clinical hypotheses: among 13 patients clinically suspected of having neuromuscular disease, 12 (92.3%) showed at least one type of peripheral neuropathy. Six patients had an electrophysiological diagnosis of peroneal neuropathy (three correctly classified clinically), and nine had polyneuropathy (five correctly classified).

Neuroimaging was performed in 36.2% of assessed cases between 2019 and 2025. Among 13 patients classified clinically as cerebrovascular disease cases, 61.5% underwent neuroimaging, with cerebrovascular lesions confirmed in 62.5% of those examined. In contrast, among 81 patients not classified as cerebrovascular disease, 32.1% underwent imaging, with relevant lesions identified in only 15.4%. No EEG reports or epilepsy clinic records were found for any patients.

Overall, these findings indicate good consistency between clinical classifications and ancillary data for neuromuscular and cerebrovascular diseases, while epilepsy diagnoses should be interpreted with caution due to the limited availability of EEG data.

### Logistic regression—WHO protocol and major diagnoses

Logistic regression models using all WHO protocol items as candidate predictors identified only a few variables significantly associated with each suspected diagnosis (Table 7). For epilepsy, loss of contact episodes were the main predictor, whereas paresthesias appeared protective.

**Table 7:** Logistic regression analysis of WHO protocol items for suspected neuromuscular disease, epilepsy, and cerebrovascular disease.

Characteristic	Neuromuscular disease			Epilepsy			Cerebrovascular disease		
	OR	95% CI	p-value	OR	95% CI	p-value	OR	95% CI	p-value
Questionnaire									
Loss of consciousness	0.82	0.24, 2.47	0.7	3.56	0.55, 17.9	0.2	2.83	0.74, 9.19	0.12
Loss of contact	0.93	0.32, 2.58	0.9	16.5	4.02, 63.0	** < 0.001**	1.02	0.23, 3.30	> 0.9
Tremors/spasms	1.04	0.53, 1.98	> 0.9	0.97	0.18, 4.59	> 0.9	0.68	0.25, 1.64	0.4
Speech impairment	0.94	0.48, 1.76	0.8	0.61	0.06, 3.36	0.6	0.9	0.38, 1.95	0.8
Facial pain	0.9	0.29, 2.67	0.9	0.64	0.00, 7.98	0.8	0.73	0.13, 2.82	0.7
Facial paralysis	0.38	0.05, 2.32	0.3	0.06	0.00, 4.62	0.2	10.2	1.53, 72.2	**0.018**
Muscle weakness	1.83	1.04, 3.20	**0.036**	1.03	0.19, 4.07	> 0.9	1.12	0.51, 2.33	0.8
Gait impairment	1.32	0.73, 2.37	0.4	1.38	0.33, 5.23	0.6	1.45	0.67, 3.05	0.3
Paresthesias	6.45	4.23, 9.91	** < 0.001**	0.13	0.01, 0.73	**0.018**	1.39	0.74, 2.54	0.3
Headache	0.8	0.53, 1.20	0.3	1.68	0.57, 4.92	0.3	0.84	0.48, 1.45	0.5
Triage physical exam									
Arms extended	1	0.32, 3.00	> 0.9	2.88	0.22, 27.6	0.4	1.45	0.42, 4.56	0.5
Match stick test	0.9	0.36, 2.22	0.8	0.8	0.03, 8.45	0.9	1.87	0.70, 4.69	0.2
Cloth sensation test	0.62	0.21, 1.72	0.4	2.79	0.29, 17.4	0.3	1.2	0.37, 3.40	0.7
Index-nose test	0.88	0.32, 2.28	0.8	1.12	0.13, 7.28	> 0.9	2.38	0.83, 6.26	0.1
Tandem walk test	3.27	1.92, 5.60	** < 0.001**	2.28	0.52, 8.49	0.3	1.74	0.85, 3.46	0.13
Balance	0.39	0.09, 1.61	0.2	1.98	0.13, > 200	0.7	1.33	0.33, 6.37	0.7
Romberg’s test	1.74	0.51, 5.88	0.4	1.24	0.01, 12.3	0.9	1.11	0.25, 3.84	0.9

Significant values are in [bold].

Odds ratios (OR), 95% confidence intervals (CI), and p-values from independent multivariable logistic regression models adjusted for all listed WHO protocol items. P-values are adjusted within but not across models and are interpreted descriptively.

Neuromuscular diseases were most strongly linked to paresthesias (OR, 6.45), and, to a lesser extent, to muscle weakness, and tandem-walk failure, while cerebrovascular disease was associated with facial paralysis only.

In the best-subset models (Table 8), tandem-walk failure was a common predictor across diagnoses, while facial paralysis was specific to cerebrovascular disease. Loss of contact episodes strongly predicted epilepsy, and paresthesias remained the principal correlate of neuromuscular disease. As the correlation of tandem walk failure and epilepsy was unanticipated, a post hoc exploratory analysis was carried out. Tandem walk test failure was found to be strongly associated with gait ataxia from the comprehensive somatic examination (Fisher’s test, OR 11.4, 95% CI 5–26), possibly related to antiseizure medication use.

**Table 8:** Logistic regression analysis of WHO protocol predictors for major neurological diagnoses (simplified models from best-subsets selection).

Simplified models from best subsets selection									
Characteristic	Cerebrovascular disease			Neuromuscular disease			Epilepsy		
	OR	95% CI	p-value	OR	95% CI	p-value	OR	95% CI	p-value
Index–nose test	3.49	1.56, 7.55	**0.003**						
Tandem walk test	2.94	1.68, 5.08	** < 0.001**	3.38	2.20, 5.20	** < 0.001**	4.11	1.49, 11.1	**0.007**
Facial paralysis	12.8	2.40, 82.5	**0.003**						
Paresthesias				7.5	5.02, 11.3	** < 0.001**	0.09	0.01, 0.45	**0.001**
Loss of contact							27	8.42, 86.9	** < 0.001**

Significant values are in [bold].

Odds ratios (OR), 95% confidence intervals (CI), and p-values from independent logistic regression models derived from best-subsets selection for each outcome.

## Discussion

This study investigates long-term neurological findings among hospitalized COVID-19 survivors, identifying a significant number of cases with suspected neuromuscular and cerebrovascular disease, with few cases of suspected epilepsy. A comprehensive neurological exam showed normal findings in most cases, except for sensory and motor abnormalities, which likely reflect the high prevalence of neuromuscular conditions in the cohort. The structured neurological consult showed a very low rate of neck stiffness, abnormal movements, and cranial nerve abnormalities. This is relevant because, although cranial nerve involvement is often reported anecdotally in COVID-19 patients, only smell, taste, and vision were systematically evaluated in those studies^36–39^ .

Using the WHO protocol for assessment, we found the symptom questionnaire component especially informative, capturing prevalent potentially new-onset symptoms such as paresthesias, gait impairment, and muscle weakness— key indicators of peripheral nervous system involvement and cerebrovascular diseases. However, the somatic examination component showed notable redundancies (as clearly shown in Supplementary Fig. 2), likely because various steps measured overlapping aspects of a common neuromuscular syndrome. Model selection allowed us to identify a streamlined five-item subset of the WHO protocol. In this simplified approach, the tandem walk test was a significant predictor across all three diagnoses—neuromuscular disease, cerebrovascular disease, and epilepsy—making it especially valuable for quickly identifying high-risk patients. Although we found no specific studies evaluating patient performance on bedside tandem walking tests, gait abnormalities are common, and include poor performance in the Timed-Up and Go Test and asymmetrical gait patterns^40,41^. Despite the strengths of the 5-items subset, the final model for epilepsy performed poorly. This was likely due to the small number of cases (19 out of 708), resulting in instability and the selection of two predictors (paresthesias and tandem walk failure) with very small marginal effects that are likely of limited clinical relevance.

Our study found a significantly higher prevalence of neurological symptoms compared to population-based studies using similar versions of the WHO protocol. In our cohort, paresthesias were present in 22% of patients, gait impairment in 15%, and muscle weakness in 15%, while population-based studies reported much lower rates for similar symptoms, such as sensory changes in limbs (3.4%) and limb weakness (2.2%)^31^. The prevalence of neuromuscular disease was also much higher in our cohort (26%) compared to 0.268% for peripheral nerve disorders in the general population^25^. Similarly, cerebrovascular disease prevalence in our study (10%) was significantly higher compared to 0.174%^42^ and 0.058%^25^ reported in population-based studies, and epilepsy prevalence (2.7%) was higher than the 0.533%^25^ to 1.23%^43^ range in those studies. Additionally, our physical examination findings, such as the tandem walk test failure rate of 18%, were much higher than the less than 2% abnormality rates observed in population-based studies^31^. These differences are likely due to both the distinct study designs and the severity of COVID-19 in our cohort. Our study focused on a hospital-based cohort of severe COVID-19 patients, and used different case ascertainment criteria.

The high prevalence of neuromuscular disease, particularly polyneuropathy, is consistent with clinical observations of widespread peripheral nerve involvement in COVID-19 survivors^44–46^. Similarly to our study, high prevalence of peripheral neuropathy symptoms have been reported both early and at three months post-infection in COVID-19 patients^46^. A high incidence of focal neuropathies and plexopathies was also found for patients requiring intensive care admission in other hospital-based studies^47–49^. Although those studies highlighted the importance of upper limb neuropathies and plexopathies, we found peroneal neuropathies to be more common. This may be related to either the clinical-only nature of our diagnostic approach, or a faster recovery of compressive upper limb neuropathies. It remains unclear if the findings of neuromuscular disorders can be solely attributed to disease specific COVID-19 infection. Factors associated with prolonged hospital stay and clinical severity may also have played a significant role in these findings.

Several limitations should be considered when interpreting our findings. First, the study sample was drawn from a single tertiary hospital and consisted primarily of patients with moderate to severe COVID-19, which may limit generalizability to other settings and to milder cases. Nearly half of eligible patients (~ 47%) were excluded, largely because they could not be reached by telephone/WhatsApp or declined participation given the length and demands of the 4–5 h multidisciplinary assessment. These real-world barriers may have biased the sample toward individuals who were more reachable, healthier, or better supported, leading to potential underestimation of neurological sequelae.

Second, reliance on self-reported symptoms and retrospective chart abstraction introduces potential recall and reporting biases. In addition, the adapted Portuguese version of the WHO screening protocol was not formally re-validated in this cohort, which we acknowledge as a methodological limitation. Although the symptom questionnaire covered three reference points (pre-COVID, at discharge, and at the 6–11-month follow-up), other clinical variables were collected cross-sectionally, limiting inference about causality, incidence, and temporal progression. Moreover, the lack of a COVID-19–negative control group restricts our ability to attribute observed symptoms and diagnoses exclusively to COVID-19, as some may instead reflect pre-existing comorbidities or the general consequences of hospitalization and critical illness.

Third, although efforts were made to standardize procedures, structured neurological examinations were carried out by physicians from different specialties, which may have introduced some inter-rater variability. In addition, diagnostic classification relied primarily on automated text parsing (regex matching) of clinical notes and was only secondarily cross-checked with sparse, opportunistically obtained ancillary data (neuroimaging or electrophysiology) outside the study protocol. Because such testing was infrequent and not systematic—particularly for epilepsy, for which no EEG or specialized clinic records were available—the resulting comparisons were descriptive and cannot be considered confirmatory. Accordingly, the diagnoses presented here should be interpreted as clinical suspicions rather than definitive disease entities. Nonetheless, the reliance on clinical impressions reflects real-world diagnostic practice and provides an ecologically valid picture of neurological assessment in post-COVID patients.

Despite these limitations, the study provides valuable, pragmatic insights into post-COVID neurological outcomes in a real-world hospital cohort.

## Conclusion

In summary, this study highlights the burden of somatic neurologic disease in COVID-19 survivors and proposes a simplified screening approach using five key items from the WHO protocol to streamline post-COVID neurological care.

### Artificial intelligence use disclosure

During the preparation of this work, the author(s) used ChatGPT and LanguageTool in order to assist with drafting, language refinement, and R coding. After using these tools, the author(s) reviewed and edited the content as needed and take(s) full responsibility for the content of the publication.

## Supplementary Information

Below is the link to the electronic supplementary material.

Supplementary Material 1

Supplementary Material 2

Supplementary Material 3

Supplementary Material 4

## Data Availability

A synthetic dataset, generated using the synthpop package^50^ in R to preserve the statistical properties of the original data while ensuring privacy, is included in the GitHub repository accompanying this paper. This dataset enables readers to reproduce the analysis without access to the sensitive original data. Textual data, however, has been suppressed for privacy and ethical reasons. We acknowledge that results obtained with the synthetic dataset may differ from those derived from the original data. Data containing individual patient characteristics can be provided upon reasonable request, subject to ethical and legal considerations.
