# Supplementary material for: Neurological symptoms and physical exam findings 6–11 months post-COVID-19: a cohort study
Source: Sci Rep. 2026 Jan 2;16:3732. doi: 10.1038/s41598-025-33779-w (PMC12852936; doi:10.1038/s41598-025-33779-w)
Supplement: Supplementary file 1 — Supplementary Material 1 [file 41598_2025_33779_MOESM1_ESM.pdf]

Supplementary Methods: Neurological Screening Protocol (English Translation)

STEP 1A — Neurological Symptoms (Questionnaire)

5. Before COVID-19, did you ever have tremors or spasms of the arms or legs that you could not control?

Options: Yes, No, Don’t know, Did not answer

6. Did this symptom occur after the illness?

Options: Yes, No, Don’t know, Not applicable

8. Is your speech normal?

Options: Yes, No, Don’t know, Did not answer

10. Before COVID-19, did you ever have episodes of pain in the face?

Options: Yes, No, Don’t know, Did not answer

11. Did this symptom occur after the illness?

Options: Yes, No, Don’t know, Not applicable

13. Before COVID-19, did your face, or part of your face, ever become paralyzed for more than 24 hours?

Options: Yes, No, Don’t know, Did not answer

14. Did this symptom occur after the illness?

Options: Yes, No, Don’t know, Not applicable

16. Before COVID-19, did you ever have weakness in your arms or legs lasting more than 24 hours?

Options: Yes, No, Don’t know, Did not answer

17. Did this symptom occur after the illness?

Options: Yes, No, Don’t know, Not applicable

19. Before COVID-19, did you ever lose the ability to walk normally?

Options: Yes, No, Don’t know, Did not answer

20. Did this symptom occur after the illness?

Options: Yes, No, Don’t know, Not applicable

22. Before COVID-19, did you have reduced sensation or abnormal sensations in your arms or legs lasting more than 24 hours?

Options: Yes, No, Don’t know, Did not answer

23. Did this symptom occur after the illness?

Options: Yes, No, Don’t know, Not applicable

25. Before COVID-19, did you ever suffer from headaches?

Options: Yes, No, Don’t know, Did not answer

26. Did this symptom occur after the illness?

Options: Yes, No, Don’t know, Not applicable

STEP 1B — Neurological Examination

(Note: A bolded response indicates need for detailed evaluation in Step 2.)

Motor Tasks:

- Raise both arms above head for 30 seconds (Able, Impossible to test, Refuses, Did not understand, Unable right, Unable left, Unable bilateral)

- Pick up a stick from the floor (same response options)

Sensory/Coordination Screening:

- Cloth texture test (smooth/rough)

- Finger-to-nose test (Able, Poor control right/left/bilateral)

Gait/Balance:

- Heel-to-toe walk (Success, Unable, etc.)

- Feet together stance

STEP 2 — Detailed Neurological Assessment (Neurologist)

0. Brief History (2 lines max)

1. Motor Function:

- Motor exam: Normal, Hemiparesis, Paraparesis, Tetraparesis, Other

- Pyramidal signs: Present/Absent

- Reflexes: Hyperactive, Normal, Hypoactive, Absent

- Free-text description

2. Involuntary Movements:

- Absent, Tremor, Myoclonic, Parkinsonism, Others

- Free-text description

3. Superficial Sensation:

- Normal, Hypoesthesia patterns, Others

- Free-text description

4. Deep Sensation:

- Vibration testing: Normal or Hypopalesthesia patterns
- Free-text description

5. Coordination:

- Normal, Cerebellar hemiataxia, Global ataxia, Sensory ataxia, Others
- Free-text description

6. Meningeal Signs:

- Present/Absent

7. Cranial Nerves:

- Visual fields (II), Ocular motility, Trigeminal, Facial, Vestibulocochlear, IX–XII
- Each with structured options and free-text fields

8. Neurological Diagnoses:

- Normal, Epilepsy, Cerebrovascular disease, Polyneuropathy/Myopathy, Others
- Free-text description

Interviewer comments: Free-text field for additional post-COVID symptoms.

HOSPITAL DAS CLÍNICAS
DA FACULDADE DE MEDICINA
DA UNIVERSIDADE DE SÃO PAULO

Post-Acute Sequelae of Sars-CoV-2 (PASC): protocol for a multidisciplinary prospective observational evaluation of a cohort of patients surviving hospitalization in São Paulo, Brazil

INTERVIEW GUIDES

NEUROLOGIA

ETAPA 1a: Questionário de Sintomas

Data da avaliação [ ] Today D-M-Y

Tempo (dias) da admissão hospitalar até a realização da avaliação [ ]

Nome do médico [ ]

Para qualquer pergunta em que a resposta corresponder ao campo em negrito e sublinhado, o(a) paciente deverá fazer a avaliação neurológica mais aprofundada (ETAPA 2).

1. Você já teve perda de consciência antes da COVID-19?

- ○ **<u>Sim (mais de uma vez)</u>**
- ○ Sim (uma vez)
- ○ Nunca
- ○ Não sabe
- ○ Não respondeu

reset

2. Este sintoma aconteceu depois da doença?

- ○ **<u>Sim (mais de uma vez)</u>**
- ○ Sim (uma vez)
- ○ Nunca
- ○ Não sabe
- ○ Não se aplica

reset

3. Você já teve episódios em que perdeu o contato com o mundo ambiente antes da COVID-19?

- ○ **<u>Sim</u>**
- ○ Possível
- ○ Nunca
- ○ Não sabe
- ○ Não respondeu

reset

4. Este sintoma aconteceu depois da doença?

- ○ **<u>Sim</u>**
- ○ Possível
- ○ Nunca
- ○ Não sabe
- ○ Não se aplica

reset

5. Você já teve tremores ou espasmos dos braços e pernas que não conseguia controlar antes da COVID-19?

- **Sim**
- Não
- Não sabe
- Não respondeu

reset

6. Este sintoma aconteceu depois da doença?

- **Sim**
- Não
- Não sabe
- Não se aplica

reset

8. Sua fala é normal?

- Sim
- **Não**
- Não sabe
- Não respondeu

reset

10. Você já teve episódios de dor no rosto ou face antes da COVID-19?

- **Sim**
- Não
- Não sabe
- Não respondeu

reset

11. Este sintoma aconteceu depois da doença?

- **Sim**
- Não
- Não sabe
- Não se aplica

reset

13. Seu rosto ou face ou parte deles já ficaram paralisados mais de 24h antes da COVID-19?

- **Sim**
- Não
- Não sabe
- Não respondeu

reset

14. Este sintoma aconteceu depois da doença?

- **Sim**
- Não
- Não sabe
- Não se aplica

reset

16. Você já teve fraqueza nos seus braços ou pernas, durando mais de 24h antes da COVID-19?

- **Sim**
- Não
- Não sabe
- Não respondeu

reset

17. Este sintoma aconteceu depois da doença?

- **Sim**
- Não
- Não sabe
- Não se aplica

reset

19. Você já ficou sem conseguir andar normalmente antes da COVID-19?

- ◯ **Sim**
- ◯ Não
- ◯ Não sabe
- ◯ Não respondeu

reset

20. Este sintoma aconteceu depois da doença?

- ◯ **Sim**
- ◯ Não
- ◯ Não sabe
- ◯ Não se aplica

reset

22. Você já teve sensação diminuída, ou sensação anormal nos seus braços ou pernas, durando mais de 24h antes da COVID-19?

- ◯ **Sim**
- ◯ Não
- ◯ Não sabe
- ◯ Não respondeu

reset

23. Este sintoma aconteceu depois da doença?

- ◯ **Sim**
- ◯ Não
- ◯ Não sabe
- ◯ Não se aplica

reset

25. Você já sofreu com dor de cabeça antes da COVID-19?

- ◯ Sim
- ◯ Não
- ◯ Não sabe
- ◯ Não respondeu

reset

26. Este sintoma aconteceu depois da doença?

- ◯ Sim
- ◯ Não
- ◯ Não sabe
- ◯ Não se aplica

reset

ETAPA 1B: Exame Neurológico

Obs: Para qualquer exame em que a resposta corresponder ao campo em negrito e sublinhado, o paciente deverá fazer a avaliação neurológica mais aprofundada (ETAPA 2).

Fique com os braços acima da cabeça por 30 segundos

- ◯ Consegue ambos os membros
- ◯ Impossível testar
- ◯ Recusa
- ◯ Não entendeu
- ◯ **Incapaz lado direito**
- ◯ **Incapaz lado esquerdo**
- ◯ **Incapaz bilateral**

reset

Pegue palito do chão

- Consegue ambos os membros
- Impossível testar
- Recusa
- Não entendeu
- **Incapaz lado direito**
- **Incapaz lado esquerdo**
- **Incapaz bilateral**

reset

Feche os olhos. Sinta o pano. É liso ou áspero?

- Consegue ambos os membros
- Impossível testar
- Recusa
- Não entendeu
- **Incapaz lado direito**
- **Incapaz lado esquerdo**
- **Incapaz bilateral**

reset

Ponha as mãos na sua frente. Feche os olhos. Encoste o indicador no nariz. Repita com o outro braço

- Consegue ambos os membros
- Impossível testar
- Recusa
- Não entendeu
- **Controle ruim à direita**
- **Controle ruim à esquerda**
- **Controle ruim bilateral**

reset

Ande colocando o calcanhar encostado na ponta do pé (2 metros)

- Sucesso
- Impossível testar
- Recusa
- Não entendeu
- **Fica em pé com dificuldade**
- **Incapaz**

reset

Fique em pé com os dois pés juntos

- Sucesso
- Impossível testar
- Recusa
- Não entendeu
- **Incapaz**

reset

ETAPA 2: Avaliação Neurológica aprofundada

Esta etapa consiste em exame detalhado feito pelo neurologista.

0. Anamnese breve (explicar sintomas neurológicos pré e pós-COVID), duas linhas no máximo

Expand

1a. Motricidade - velocidade de movimentos, desvio pronador, manobras de oposição (se necessario), reflexos

- ☐ Normal
- ☐ Hemiparesia esquerda
- ☐ Hemiparesia direita
- ☐ Paraparesia
- ☐ Tetraparesia
- ☐ Outras alterações

1b. Sinais piramidais

○ Ausentes ○ Presentes

reset

1c. Reflexos

○ Exaltados ○ Presentes ○ Hipoativos ○ Abolidos

reset

1d. Descrição livre da motricidade (CAMPO LIVRE)

Expand

2a. Movimentos involuntários

- ☐ Ausentes
- ☐ Tremor
- ☐ Mioclониais
- ☐ Parkinsonismo
- ☐ Outros

2b. Descrição livre dos movimentos involuntários (CAMPO LIVRE)

Expand

3a. Sensibilidade térmico-dolorosa (alfinete)

- ☐ Normal
- ☐ Hipoestesia à direita
- ☐ Hipoestesia à esquerda
- ☐ Hipoestesia - nível
- ☐ Hipoestesia - padrão bota e luva
- ☐ Outros

2b. Descrição livre da sensibilidade superficial (CAMPO LIVRE)

Expand

4a. Palestesia (diapasão)

- ☐ Normal
- ☐ Hipopalestesia à direita
- ☐ Hipopalestesia à esquerda
- ☐ Hipopalestesia - nível
- ☐ Hipoestesia - padrão bota e luva
- ☐ Outros

4b. Descrição livre da sensibilidade profunda (CAMPO LIVRE)

Expand

5a. Coordenação

- ☐ Normal
- ☐ Hemiataxia cerebelar direita
- ☐ Hemiataxia cerebelar esquerda
- ☐ Ataxia cerebelar global
- ☐ Ataxia sensitiva
- ☐ Outros

5b. Descrição livre da coordenação (CAMPO LIVRE)

Expand

6. Sinais meníngeos (rigidez da nuca, Brudzinski)

○ Ausente ○ Presente

reset

7. Nervos cranianos

7a. II (campos visuais somente)

- ☐ Normais
- ☐ Hemianopsia (ou quadrantanopsia) direita
- ☐ Hemianopsia (ou quadrantanopsia) esquerda
- ☐ Amaurose bilateral
- ☐ Outros

7a-2. Descrição livre dos campos visuais (CAMPO LIVRE)

Expand

7b. Motricidade ocular

○ Normal ○ Paralisia de nervos cranianos

reset

7b-2. Descrição livre da motricidade ocular (CAMPO LIVRE)

Expand

7c. Trigemios

- ○ Normais
- ○ Hipoestesia direita
- ○ Hipoestesia esquerda
- ○ Outros

reset

7c-2. Descrição livre do exame do trigêmio (CAMPO LIVRE)

Expand

7d. Faciais

- ☐ Normais
- ☐ Paralisia facial direita - padrão periférico
- ☐ Paralisia facial direita - padrão central
- ☐ Paralisia facial esquerda - padrão periférico
- ☐ Paralisia facial esquerda - padrão central
- ☐ Outros

7d-2. Descrição livre de nervos faciais (CAMPO LIVRE)

Expand

7e. Vestíbulo-cocleares (nistagmo, VOR, não testar audição)

- ○ Normais
- ○ Disfunção à direita
- ○ Disfunção à esquerda
- ○ Outros

reset

7e-2. Descrição livre de nervos vestíbulo-cocleares (CAMPO LIVRE)

Expand

7f. Glossofaríngeo, Vago, Acessorio, Hipoglosso (IX-XII)

○ Normais ○ Alterados

reset

7f-2. Descrição livre de nervos IX-XII (CAMPO LIVRE)

Expand

8a. Diagnósticos neurológicos (mais de uma possibilidade)

- ☐ Paciente normal
- ☐ Epilepsia
- ☐ Doença cerebrovascular
- ☐ Polineuropatia/miopatia
- ☐ Outros

8b. Descrição livre dos diagnósticos neurológicos (CAMPO LIVRE)

Expand

Entrevistador(a): no espaço abaixo, registrar informações adicionais sobre sintomas apresentados pelo paciente após a internação por COVID-19.

Expand
