# Supplementary material for: Neurological symptoms and physical exam findings 6–11 months post-COVID-19: a cohort study
Source: Sci Rep. 2026 Jan 2;16:3732. doi: 10.1038/s41598-025-33779-w (PMC12852936; doi:10.1038/s41598-025-33779-w)
Supplement: Supplementary file 2 — Supplementary Material 2 [file 41598_2025_33779_MOESM2_ESM.pdf]

Supplemental file 2

Post-hoc classification of potential clinical diagnoses

Corresponding files in the GitHub repository:

`/data_wrangling/recode_diagnosis_neuromuscular.R`

`/data_wrangling/recode_diagnosis_epilepsy.R`

`/data_wrangling/recode_cerebrovascular.R`

`/data_wrangling/recode_diagnosis_miscelaneous.R`

These files are sourced from the "/data_wrangling/main_wrangling_script.R" file, which builds the analytical dataset from the raw data.

Pattern matching was conducted using regular expressions (regex) implemented in R. The pattern sets were derived through an iterative refinement process based on clinical expertise and review of the dataset. All diagnoses were assigned based on analysis of free-text information extracted from the capture fields "diagnostico – descrição" (diagnosis – description).

Neuromuscular disorder diagnosis criteria

A diagnosis of neuromuscular disorder was given when text matched any of the following strings or substrings:

Required matching

"radicu", "neurop", "mononeuro", "fibra", "nervo", "femo", "fibul", "meral", "carpo", "mediano", "ulnar", "fraqueza distal", "fraqueza muscular", "poline", "neurite", "miopa", "neurofibromatose", "lateral amio", "\bmg\b", "\bpnp\b", "perif[eé]rica", "luva", "bota", "quatro membros", "comprometimento muscular", "plexo", "sensitiva distal"

Exclusion criteria (did not match)

"assintom", "sem sintoma", "sinais leves", "subcl[ií]", "sem nenhuma outra", "sem outros achados de", "j[aAáÁ] resolv", "sem polineuropatia", "funcional", "talvez", "duvidos", "dif[ií]cil", "sutil", "inconsistente", "question.vel", "neuropatia//?", "neurite vestibu", "mas n[aã]o o exame", "formigamento mao es", "paralisia facial", "neuropatia vest[íi]bu"

Classify subtypes of neuromuscular disorders

Polyneuropathy

Required matching

"pol[ií][mn]e", "pnp", "fibra", "bota", "sensitivo", "diab[ée]tica", "tetraparesia", "perif[ée]", "tetra.*bilat.*assim", "gradiente sensitivo mmii", "força nos quatro membros", "comprimento de"

Exclusion criteria (did not match)

"sensibilidade mi[ed] em bota"

Mononeuropathies

Fibular neuropathy

Required matching

"fibular"

Lateral femoral neuropathy

Required matching

"meralg", "lateral.*cox"

Other lower limb neuropathies

Required matching

"fem[uo]", "\btibia", "sci[aá]t", "mononeuropatia (mie|membro)"

Median neuropathy

Required matching

"mediano", "carpo", "t[uú]nel"

Ulnar neuropathy

Required matching

"ulnar", "cotovel"

Other upper limb neuropathies

Required matching

"radial", "axilar", "mú[u]sculo.{1}cut[aâá]"

Motor neuron disease

Required matching

"neur[oôó]nio.{1}motor", "\bela\b", "amiotr[óo]f", "muscular espinh"

Radiculopathy

Required matching

"rad[ií]culo"

Exclusion criteria (did not match)

"rad[ií]culo.*\/neuro", "\?"

Epilepsy diagnosis criteria

A diagnosis of epilepsy was given when text matched any of the following strings or substrings:

Required matching

"epil[eé]", "carba", "levetir", "levotir", "fenob", "fenito", "hidan", "olhar fixo", "epis[oó]dios.*estereotipados"

Exclusion criteria (did not match)

"sem crise", "epilepsia.*resolvida"

Cerebrovascular disease diagnosis criteria

Because the clinical diagnosis of cerebrovascular disease is elusive, the diagnosis of cerebrovascular disease was based on the presence of ANY of the following:

-pattern matching of either the capture field “diagnostico – descrição” (diagnosis – description) or the capture filed “anamnese” (anamnesis), as outlined below.

-A history of stroke as extracted from their hospital admission charts

Pattern matching:

A diagnosis of cerebrovascular disease was given when text matched any of the following strings or substrings:

Required matching

"avc", "ait", "\bave\b", "isquemia.{0,3}cerebral", "hemiparesia", "aneurisma.{0,3}", "\bhsa\b", "hemi.{0,2}hi.*poestesia alterna", "lacunar", "hemiparesia", "afasia", "evento central", "hipoestesia (e(s|)|d(ir|)|comple)", "cerebrovascular"

Exclusion criteria (did not match)

"hipoestesia e parestesia dolorosa distal", "hipoestesia e dor", "funcional", "encefalopatia", "mielopatia", "cervical pr[ée]via", "parece ser do trauma", "oportunista", "infec[cç][aã] oportu", "ortop", "princ[ií]pio", "aneurisma de aorta", "pseudoaneurismas", "\bhiv\b", "tce", "mening[ie]oma", "amn[ée]sia lacunar", "rm sem les", "astrocit", "tumor", "não visualizad.{0,4} na consulta", "déficit sensorial mie.* doença cerebrovascular", "neurocirurgia", "cirurgia em coluna"

Miscellaneous diagnoses criteria

Parkinsonism

Required matching

"park"

Tremor

Required matching

"tremor"

Exclusion criteria (did not match)

"park", "funcional", "ansied", "dissoci", "atax"
