# Supplementary material for: Neurological symptoms and physical exam findings 6–11 months post-COVID-19: a cohort study
Source: Sci Rep. 2026 Jan 2;16:3732. doi: 10.1038/s41598-025-33779-w (PMC12852936; doi:10.1038/s41598-025-33779-w)
Supplement: Supplementary file 3 — Supplementary Material 3 [file 41598_2025_33779_MOESM3_ESM.html]

Neurological Symptoms and Physical Exam Findings 6–11 Months Post-COVID-19: A Cohort Study


# Neurological Symptoms and Physical Exam Findings 6–11 Months Post-COVID-19: A Cohort Study

Main analysis script with reproducible workflow

Author

Guedes BF et al

# Setup

```
# Definir funções personalizadas a usar ao longo da analise
my_percent <- \(x) scales::label_percent(accuracy = 0.1)(x)

custom_median <- function(x) stats::median(x, na.rm = TRUE)
```

```
set.seed(2025)
```

# Data loading

```
# set option to keep id columns
keep_id_columns <- TRUE

df                <- import(here("analytical_data", "analytical_data.csv"), setclass = "tibble")
df_neuromuscular  <- import(here("analytical_data", "neuromuscular_diagnoses.csv"), setclass = "tibble")
df_epilepsy       <- import(here("analytical_data", "epilepsy_diagnoses.csv"), setclass = "tibble")
df_cerebrovascular<- import(here("analytical_data", "cerebrovascular_diagnoses.csv"), setclass = "tibble")
df_other_diagnoses<- import(here("analytical_data", "other_diagnoses.csv"), setclass = "tibble")

if(keep_id_columns == FALSE){
    df <- select(df, -c(record_id, medical_record))
    df_neuromuscular <- select(df_neuromuscular, -c(record_id, medical_record))
    df_epilepsy <- select(df_epilepsy, -c(record_id, medical_record))
    df_cerebrovascular <- select(df_cerebrovascular, -c(record_id, medical_record))
    df_other_diagnoses <- select(df_other_diagnoses, -c(record_id, medical_record))
}


# data.frame with the three most relevant diagnoses
diagnoses <- 
    bind_cols(`Neuromuscular_disease` = df_neuromuscular$diagnosis_neuromuscular |> 
                  as.factor(),
              `Epilepsy` = df_epilepsy$diagnosis_epilepsy |> 
                  as.factor(),
              `Cerebrovascular_disease` = df_cerebrovascular$diagnosis_cerebrovascular |> 
                  as.factor())
```

# Table 1

```
table_1_baseline <- df |>
    select(
        age,
        sex,
        diabetes,
        hypertension,
        chronic_kidney_disease,
        stroke,
        myocardial_infarction,
        bmi_admission,
        icu_care
    ) |>
    mutate(icu_care = ifelse(icu_care,
                             'ICU-admitted',
                             'ICU non-admitted')) |>
    tbl_summary(
        by = icu_care,
        statistic = list(
            all_continuous() ~ "{custom_median} [{p25} - {p75}]",
            all_categorical() ~ "{n} ({p}%)"
        ),
        value = sex ~ "female",
        label = list(
            age ~ "Age",
            sex ~ "Sex - female",
            diabetes ~ "Diabetes",
            hypertension ~ "Hypertension",
            chronic_kidney_disease ~ "Chronic kidney disease",
            stroke ~ "Previous cerebrovascular disease",
            myocardial_infarction ~ "Previous myocardial infarction",
            bmi_admission ~ "Body mass index"
        ),
        missing = "no"
    ) |>
    # add_p(test = list(all_categorical() ~ 'fisher.test',
                      # all_continuous() ~ 'wilcox.test')) |>
    modify_header(label ~ "") |>
    # modify_caption("**Patient Characteristics**") |>
    modify_column_indent(columns = label, double_indent = TRUE)
    
table_1_acute_infection <- df |>
    select(los_total,
           icu_care,
           icu_los_2,
           intubation) |>
    mutate(icu_care = ifelse(icu_care, 'ICU-admitted', 'ICU non-admitted')) |> 
    tbl_summary(by = icu_care,
                statistic = list(all_continuous() ~ "{custom_median} [{p25} - {p75}]",
                                 all_categorical() ~ "{n} ({p}%)"),
                label = list(los_total ~ "Length of hospital stay",
                             # icu_care ~ "Intensive care admission",
                             icu_los_2 ~ "Intensive care - lenght of stay",
                             intubation ~ "Intubation"),
                missing = "no"
                ) |>
    modify_header(label ~ "") |>
    # modify_caption("**Table 1. Patient Characteristics**") |>
    modify_column_indent(columns = label, double_indent = TRUE)

table_1_list <- list(table_1_baseline, table_1_acute_infection)

baseline_table <- tbl_stack(table_1_list,
          group_header = c("Baseline characteristics",
                           "Acute COVID characteristics"),
          # quiet = NULL
          )

baseline_table <- tbl_stack(table_1_list,
          group_header = c("Baseline characteristics",
                           "Acute COVID characteristics"),
          # quiet = NULL
          ) |> 
  as_gt() |> 
  tab_header(
    title = md("**Table 1 - Baseline and Acute COVID characteristics**"))
baseline_table
```

| **Table 1 - Baseline and Acute COVID characteristics** | | |
| --- | --- | --- |
|  | **ICU non-admitted**  N = 3011 | **ICU-admitted**  N = 4071 |
| Baseline characteristics | | |
| Age | 56 [44 - 66] | 57 [44 - 65] |
| Sex - female | 143 (48%) | 192 (47%) |
| Diabetes | 97 (32%) | 151 (37%) |
| Hypertension | 164 (55%) | 237 (58%) |
| Chronic kidney disease | 40 (14%) | 25 (6.3%) |
| Previous cerebrovascular disease | 11 (3.7%) | 20 (4.9%) |
| Previous myocardial infarction | 25 (8.4%) | 38 (9.6%) |
| Body mass index | 29 [26 - 34] | 28 [24 - 34] |
| Acute COVID characteristics | | |
| Length of hospital stay | 6 [4 - 9] | 20 [13 - 33] |
| Intensive care - lenght of stay | NA [NA - NA] | 10 [6 - 18] |
| Intubation | 1 (0.3%) | 275 (68%) |
|  |  |  |
| --- | --- | --- |
| 1 custom\_median [Q1 - Q3]; n (%) | | |

# wrangling of who protocol data

```
# data.frame of who physical examination steps
who_physical <- 
    df |> 
    select(starts_with("who"), -ends_with("reason"))

# data.frame of who questions steps
who_symptoms <- 
    df |>
    select(matches("Q.*fup"))

# extract names of the diagnoses columns for the glm formulas
diagnoses_names <- colnames(diagnoses)

#extract names of the predictors for the formulas
predictors_names <- c(colnames(who_physical),
                      colnames(who_symptoms))

# create the analytical dataset for who protocol findings
who_vs_diagnoses <- 
    bind_cols(who_physical,
              who_symptoms,
              diagnoses)

# preprocess the dataset
# Abnormal findings in the predictors and positive outcomes are set to TRUE,
# all other values, including NAs, are set to FALSE

who_vs_diagnoses <- 
    who_vs_diagnoses |> 
    mutate(across(starts_with("Q"),
                  \(x) if_else(x %in% "yes", TRUE, FALSE)),
           across(starts_with("who"),
                  \(x) if_else(x %in% "normal", FALSE, TRUE)))
```

# WHO questionnaire

```
##############################################################
##            NEUROLOGICAL SYMPTOM ANALYSIS SCRIPT           ##
##############################################################

# Helper
my_percent <- scales::label_percent(accuracy = 0.1)

##################################################################
##                         DATA WRANGLING                       ##
##################################################################

# Define both long and short names
question_labels <- list(
    long = list(
        Q1  = "Loss of consciousness",
        Q2  = "Loss of contact",
        Q3  = "Tremors or spasms",
        Q4  = "Speech impairment",
        Q5  = "Facial paralysis",
        Q6  = "Facial pain",
        Q7  = "Muscle weakness",
        Q8  = "Gait impairment",
        Q9  = "Paresthesias",
        Q10 = "Headache"
    ),
    short = list(
        Q1  = "LOC",
        Q2  = "Contact",
        Q3  = "Tremors",
        Q4  = "Speech",
        Q5  = "Face paral",
        Q6  = "Face pain",
        Q7  = "Weakness",
        Q8  = "Gait",
        Q9  = "Paresth",
        Q10 = "Headache"
    )
)

# Subset to questionnaire items
questions <- df |>
    select(matches("^Q\\d+")) |>
    # keep only before / after / follow-up questions
    select(matches("(fup|after|before)$"))

# Long format with labels + stable ordering
questions_long <- questions |>
    mutate(index = factor(row_number()), .before = everything()) |>
    pivot_longer(-index, names_to = "complex_name", values_to = "value") |>
    mutate(
        question = str_extract(complex_name, "Q\\d+"),
        value    = factor(if_else(value %in% "yes", "yes", "no")),
        timing   = str_extract(complex_name, "before|after|fup"), #|>
        # str_replace("fup", "follow-up"),
        timing   = factor(timing, levels = c("before", "after", "fup")),
        question = factor(question, levels = paste0("Q", 1:10)),
        question_long  = recode(as.character(question), !!!question_labels$long),
        question_short = recode(as.character(question), !!!question_labels$short),
        question_pretty_long  = glue("{question_long} {timing}"),
        question_pretty_short = glue("{question_short} {timing}")
    ) |>
    droplevels() |> 
    arrange(question, timing)

# question rates
questions_rates <- questions_long |>
    group_by(question, question_pretty_short, timing) |>
    summarise(
        rate = mean(value == "yes", na.rm = TRUE),
        rate_pct = my_percent(rate),
        .groups = "drop"
    )

##################################################################
##                        Mcnemars tests                     ##
##################################################################

questions_tests <- questions_long |>
  filter(timing %in% c("before", "fup")) |>  
    droplevels() |> 
    pivot_wider(names_from = timing, 
                values_from = value,
                id_cols = c(index, question)) |> 
  group_by(question) |> 
    nest() |> 
    ungroup()

questions_tests <- 
    questions_tests |>
    rowwise() |>
    mutate(tab = list(table(before = data$before,
                            fup = data$fup)),
           one_way_mcnemar = list(exact2x2(tab,
                                         alternative = "greater",
                                         paired = TRUE)),
           one_way_mcnemar = pluck(one_way_mcnemar, "p.value"))

##################################################################
##     TABLE WITH rates and mcnemars ##
##################################################################

# 1. Prevalence rates (wide)
questions_summary_wide <- questions_rates |>
    select(question, timing, rate_pct) |>
    pivot_wider(
        names_from  = timing,
        values_from = rate_pct
    ) |>
    mutate(
        question_label = recode(as.character(question), !!!question_labels$long)
    ) |>
    select(question, question_label, before, after, fup)

questions_summary_wide <-
    questions_summary_wide |> 
    left_join(select(questions_tests, c(question, one_way_mcnemar)),
                     by = "question")

# adjust p values:

questions_summary_wide <-
    questions_summary_wide |> 
    mutate(one_way_mcnemar = p.adjust(one_way_mcnemar, method = "fdr"))

symptom_table_gt <- questions_summary_wide |>
  select(question_label, before, after, fup, one_way_mcnemar) |>
  gt(rowname_col = "question_label") |>
  tab_header(
    title = md("**Symptom Prevalence and One-Way McNemar Tests**"),
    subtitle = md("Before COVID, At Discharge, and Follow-up")
  ) |>
  cols_label(
    before          = md("**Before COVID**"),
    after           = md("**At Discharge**"),
    fup             = md("**Follow-up**"),
    one_way_mcnemar = md("**Exact McNemar (p)**")
  ) |>
  fmt(
    columns = one_way_mcnemar,
    fns = scales::label_pvalue(accuracy = 0.001, add_p = FALSE)
  ) |>
  fmt_missing(columns = everything(), missing_text = "–") |>
  tab_options(table.font.size = px(12))

symptom_table_gt
```

| **Symptom Prevalence and One-Way McNemar Tests** | | | | |
| --- | --- | --- | --- | --- |
| Before COVID, At Discharge, and Follow-up | | | | |
|  | **Before COVID** | **At Discharge** | **Follow-up** | **Exact McNemar (p)** |
| Loss of consciousness | 7.2% | – | 3.2% | >0.999 |
| Loss of contact | 6.5% | – | 3.8% | >0.999 |
| Tremors or spasms | 8.1% | 14.1% | 9.7% | 0.195 |
| Speech impairment | 5.8% | – | 10.7% | <0.001 |
| Facial paralysis | 3.0% | 4.5% | 3.4% | 0.607 |
| Facial pain | 4.8% | 1.1% | 1.0% | >0.999 |
| Muscle weakness | 7.6% | 28.1% | 15.5% | <0.001 |
| Gait impairment | 9.9% | 33.6% | 15.3% | 0.001 |
| Paresthesias | 11.7% | 26.4% | 22.0% | <0.001 |
| Headache | 41.0% | – | 40.3% | 0.975 |

# WHO physical examination

```
# labels

physical_labels <- list(
  "Arms extended"             = "who_arms_extended",
  "Match stick pick-up"       = "who_match_stick",
  "Cloth discrimination test" = "who_cloth_gross_sensation",
  "Index-nose test"           = "who_index_nose",
  "Tandem walk test"          = "who_tandem",
  "Balance test"              = "who_balance",
  "Romberg's test"            = "who_romberg"
)

#relabel
who_physical <- who_physical |> 
    rename(
        !!!physical_labels
    )

#table
tbl_summary(who_physical) |> 
    as_gt() |> 
    tab_header(title = md('**Physical examination findings**'),
               subtitle = md('WHO protocol - step 2'))
```

| **Physical examination findings** | |
| --- | --- |
| WHO protocol - step 2 | |
| **Characteristic** | **N = 708**1 |
| Arms extended |  |
| bilateral deficit | 6 (0.9%) |
| normal | 678 (96%) |
| unilateral deficit | 20 (2.8%) |
| Unknown | 4 |
| Match stick pick-up |  |
| bilateral deficit | 11 (1.6%) |
| normal | 658 (95%) |
| unilateral deficit | 22 (3.2%) |
| Unknown | 17 |
| Cloth discrimination test |  |
| bilateral deficit | 8 (1.1%) |
| normal | 680 (97%) |
| unilateral deficit | 13 (1.9%) |
| Unknown | 7 |
| Index-nose test |  |
| bilateral deficit | 10 (1.4%) |
| normal | 670 (96%) |
| unilateral deficit | 17 (2.4%) |
| Unknown | 11 |
| Tandem walk test |  |
| complete deficit/incapable | 91 (13%) |
| normal | 567 (82%) |
| partial deficit/difficulty | 33 (4.8%) |
| Unknown | 17 |
| Balance test |  |
| complete deficit/incapable | 23 (3.3%) |
| normal | 673 (97%) |
| Unknown | 12 |
| Romberg's test |  |
| complete deficit/incapable | 11 (1.6%) |
| could\_not\_stand | 1 (0.1%) |
| normal | 657 (98%) |
| Unknown | 39 |
|  |  |
| --- | --- |
| 1 n (%) | |

# WHO protocol correlation plot

```
##################################################################
##               Cramer's V - association heatmap               ##
##################################################################

# Prepare the data.frame with only predictor variables
predictors_data.frame <- 
  who_vs_diagnoses |> 
  select(all_of(predictors_names))

# Function to compute Cramér's V for each pair of binary variables
cramers_v <- function(x, y) {
  tbl <- table(x, y)
  assocstats(tbl)$cramer
}

# Calculate the Cramér's V matrix
cramer_matrix <- outer(predictors_data.frame, predictors_data.frame, Vectorize(cramers_v))
rownames(cramer_matrix) <- colnames(cramer_matrix) <- predictors_names

renaming_vector <- c(
  "who_arms_extended" = "Arms extended", 
  "who_match_stick" = "match stick test",
  "who_cloth_gross_sensation" = "cloth sensation test",
  "who_index_nose" = "index-nose test",
  "who_tandem" = "tandem walk test",
  "who_balance" = "balance",
  "who_romberg" = "Romberg's test",
  "Q1_loc.fup" = "Loss of consciousness",
  "Q2_contact.fup" = "Loss of contact",
  "Q3_tremors_spasms.fup" = "Tremors/spasms",
  "Q4_speech.fup" = "Speech impairment",
  "Q5_face_pain.fup" = "Facial pain",
  "Q6_face_paral.fup" = "Facial paralysis",
  "Q7_weakness.fup" = "Muscle weakness",
  "Q8_walk.fup" = "Gait impairment",
  "Q9_paresthesia.fup" = "Paresthesias",
  "Q10_headache.fup" = "Headache"
)

# Apply renaming to the row and column names
rownames(cramer_matrix) <- renaming_vector[rownames(cramer_matrix)]
colnames(cramer_matrix) <- renaming_vector[colnames(cramer_matrix)]

# The cramer_matrix now has the renamed rows and columns

# enlarge text downstream
par(cex = 1.4)

# plot the corrplot
corrplot_who <- 
corrplot(
    cramer_matrix,
    method = 'circle',
    col = COL2("RdYlBu", 10),
    tl.col = "black",
    col.lim = c(0, 1),
    title = "Association Matrix (Cramér's V) - WHO Protocol",
    mar=c(0,0,3,0)
)

corrRect(corrplot_who,
         name = c("Arms extended",
                  "Loss of consciousness",
                  "Headache")
         )
```

```
# text size back to default
par(cex = 1)
```

# Structured physical examination

```
# Select variables with semi-structured neurological examination findings

physical <-
    df |>
    select(c(motor,
           pinprick,
           vibration,
           coordination,
           involuntary_movements,
           nuchal_rigity,
           starts_with('cn')))

# Remove unecessary levels, simplify into 'normal/non-normal'

physical <-
    physical |>
    mutate(
        Motor = fct_relevel(
            motor,
            c(
                "monoparesis",
                "hemiparesis",
                "paraparesis",
                "quadriparesis",
                "other",
                "normal"
            )
        ),
        `Pinprick sensation` = factor(
            pinprick |>
                as.character() |>
                str_replace_all("_", " "),
            c(
                "focal abnormalities",
                "hemi hypoesthesia",
                "gradient",
                "level",
                "other",
                "normal"
            )
        ),
        Coordination = factor(
            coordination |>
                str_replace_all("_", " "),
            levels = c(
                "cerebellar ataxia",
                "sensory ataxia",
                "normal",
                "unclear/not tested"
            )
        ),
        `Vibration sense` = factor(
            vibration |>
                as.character() |>
                str_replace("_", " "),
            c(
                "focal",
                "hemi hypoesthesia",
                "gradient",
                "level",
                "other",
                "normal"
            )
        ),
        `Involuntary movements` = factor(
            involuntary_movements,
            levels = c("tremor",
                       "parkinsonism",
                       "myoclonus",
                       "other",
                       "normal")
        ),
        cn2 = factor(
            cn2 |>
                as.character() |>
                str_replace_all("_", " "),
            c(
                "unilateral vision loss",
                "hemianopia",
                "bilateral amaurosis",
                "did not understand/not tested",
                "other",
                "normal"
            )
        ),
        cn5 = factor(
            cn5 |>
                as.character() |>
                str_replace_all("_", " "),
            c(
                "unilateral dysfunction",
                "did not understand/not tested",
                "other",
                "normal"
            )
        ),

        cn7 = factor(
            cn7 |>
                as.character() |>
                str_replace_all("_", " "),
            c(
                "central dysfunction",
                "peripheral dysfunction",
                "other",
                "normal"
            )
        ),
        cn8 = factor(
            cn8 |>
                as.character() |>
                str_replace_all("_", " "),
            c(
                "unilateral dysfunction",
                "bilateral dysfunction",
                "other",
                "normal"
            )
        ),
        cn9_12 = factor(
            as.character(cn9_12) |>
                str_replace_all("_", " "),
            c("bulbar dysfunction", "normal")
        ),
        `Nuchal rigity` = case_when(
            nuchal_rigity == 1 ~ "present",
            nuchal_rigity == 0 ~ "absent",
            .default = "unclear/not tested"
        ),
        .keep = "unused"
    ) |>
    mutate(across(
        everything(),
        \(x) fct_na_value_to_level(x, "unclear/not tested")
    )) |>
    mutate(across(
        where(\(x) any(
            levels(x) == "did not understand/not tested"
        )),
        \(x) fct_recode(x, "unclear/not tested" = "did not understand/not tested")
    )) |>
    mutate(across(
        everything(),
        \(x) fct_relevel(x, "unclear/not tested", after = Inf)
    ))

# rename with pretty names

convert_to_roman <- function(name) {
    name <- str_replace(name, "(?<!\\d)2", "II")
    name <- str_replace(name, "3 6.*", "III-VI")
    name <- str_replace(name, "5", "V")
    name <- str_replace(name, "7", "VII")
    name <- str_replace(name, "8", "VIII")
    name <- str_replace(name, "9 12", "IX-XII")
    return(name)
}

physical <-
    physical |>
    rename_with(
        .fn = \(x)
        str_replace(x, "_", " ") |>
            str_replace("cn", "") |>
            str_to_sentence() |>
            convert_to_roman(
            ))

physical_list <-
    list("Cranial Nerves" = select(physical, matches("II|V\\b|VII|IX|III")),
         "Somatic examination" = select(physical, !matches("II|V\\b|VII|IX|III")))

physical_summaries <-
  physical_list |>
  map(~ tbl_summary(.x, missing = "no"))

physical_tabyls <- 
  physical |> 
  map(tabyl) |> 
  map(adorn_pct_formatting, digits = 1) |> 
  map(rename_with, \(x) "finding", 1)
```

```
as_gt(physical_summaries[[1]]) |>
  gt::tab_options(table.width = gt::pct(100)) |>
  gt::tab_header(title = md("**Cranial Nerves**"))
as_gt(physical_summaries[[2]]) |>
  gt::tab_options(table.width = gt::pct(100)) |>
  gt::tab_header(title = md("**Somatic Examination**"))
```

| **Cranial Nerves** | |
| --- | --- |
| **Characteristic** | **N = 708**1 |
| II |  |
| unilateral vision loss | 15 (2.1%) |
| hemianopia | 6 (0.8%) |
| bilateral amaurosis | 1 (0.1%) |
| other | 5 (0.7%) |
| normal | 674 (95%) |
| unclear/not tested | 7 (1.0%) |
| III-VI |  |
| normal | 699 (99%) |
| ophthalmoparesis | 9 (1.3%) |
| unclear/not tested | 0 (0%) |
| V |  |
| unilateral dysfunction | 15 (2.1%) |
| other | 3 (0.4%) |
| normal | 686 (97%) |
| unclear/not tested | 4 (0.6%) |
| VII |  |
| central dysfunction | 21 (3.0%) |
| peripheral dysfunction | 9 (1.3%) |
| other | 4 (0.6%) |
| normal | 674 (95%) |
| unclear/not tested | 0 (0%) |
| VIII |  |
| unilateral dysfunction | 6 (0.8%) |
| bilateral dysfunction | 8 (1.1%) |
| other | 2 (0.3%) |
| normal | 692 (98%) |
| unclear/not tested | 0 (0%) |
| IX-XII |  |
| bulbar dysfunction | 6 (0.8%) |
| normal | 702 (99%) |
| unclear/not tested | 0 (0%) |
|  |  |
| --- | --- |
| 1 n (%) | |

| **Somatic Examination** | |
| --- | --- |
| **Characteristic** | **N = 708**1 |
| Motor |  |
| monoparesis | 47 (6.6%) |
| hemiparesis | 44 (6.2%) |
| paraparesis | 13 (1.8%) |
| quadriparesis | 19 (2.7%) |
| other | 14 (2.0%) |
| normal | 571 (81%) |
| unclear/not tested | 0 (0%) |
| Pinprick sensation |  |
| focal abnormalities | 67 (9.5%) |
| hemi hypoesthesia | 29 (4.1%) |
| gradient | 90 (13%) |
| level | 6 (0.8%) |
| other | 17 (2.4%) |
| normal | 499 (70%) |
| unclear/not tested | 0 (0%) |
| Coordination |  |
| cerebellar ataxia | 7 (1.0%) |
| sensory ataxia | 30 (4.2%) |
| normal | 662 (94%) |
| unclear/not tested | 9 (1.3%) |
| Vibration sense |  |
| focal | 17 (2.4%) |
| hemi hypoesthesia | 12 (1.7%) |
| gradient | 111 (16%) |
| level | 1 (0.1%) |
| other | 11 (1.6%) |
| normal | 556 (79%) |
| unclear/not tested | 0 (0%) |
| Involuntary movements |  |
| tremor | 46 (6.5%) |
| parkinsonism | 5 (0.7%) |
| myoclonus | 3 (0.4%) |
| other | 0 (0%) |
| normal | 654 (92%) |
| unclear/not tested | 0 (0%) |
| Nuchal rigity |  |
| absent | 702 (99%) |
| present | 3 (0.4%) |
| unclear/not tested | 3 (0.4%) |
|  |  |
| --- | --- |
| 1 n (%) | |

# Suspected diagnoses

```
# rename with pretty names, remove irrelevant columns
df_neuromuscular_2 <-
    df_neuromuscular |>
    select(-c(medical_record, record_id)) |>
    select(-matches("euromuscular.*ssified")) |>
    relocate(any_specified_mono, .before = matches("fibular_neuropathy")) |>
    rename(
        "Any neuromuscular disease" = diagnosis_neuromuscular,
        "Any mononeuropathy" = "any_specified_mono",
        "peroneal neuropathy" = fibular_neuropathy,
        "Motor neuron disease" = motor_neuron
    ) |>
    rename_with(\(x) str_replace_all(x, "_", " ") |> str_to_sentence())

df_cerebrovascular_2 <-
    df_cerebrovascular |>
    select(-c(medical_record, record_id)) |>
    rename("Cerebrovascular disease" = "diagnosis_cerebrovascular")

df_epilepsy_2 <-
    df_epilepsy |>
    select(-c(medical_record, record_id)) |>
    rename("Epilepsy" = "diagnosis_epilepsy")

df_other_diagnoses_2 <-
    df_other_diagnoses |>
    select(-c(medical_record, record_id)) |>
    rename_with(.cols = everything(), \(x) str_to_sentence(x))

neuromuscular_table <-
    df_neuromuscular_2 |>
    tbl_summary(missing = "no") |>
    modify_table_body(\(x) filter(x, stat_0 != "0 (0%)")) |>
    bstfun::add_variable_grouping(
        "Mononeuropathies" = c(
            "Any mononeuropathy",
            "Peroneal neuropathy",
            "Lateral femoral neuropathy",
            "Other lower limb neuropathies",
            "Median neuropathy",
            "Ulnar neuropathy",
            "Other upper limb neuropathies"
        )
    ) |>
    modify_header()

cerebrovascular_table <-
    df_cerebrovascular_2 |>
    tbl_summary()

epilepsy_table <-
    df_epilepsy_2 |>
    tbl_summary()

other_diagnoses_table <-
    df_other_diagnoses_2 |>
    tbl_summary()

tbl_stack(
    tbls = list(
        neuromuscular_table,
        cerebrovascular_table,
        epilepsy_table,
        other_diagnoses_table
    ),
    group_header = c(
        "Neuromuscular disease",
        "Cerebrovascular disease",
        "Epilepsy",
        "Other diagnoses"
    )
) |>
    modify_header(label ~ "**Condition**") |>
    as_gt() |>
    gt::tab_style(
        style = gt::cell_text(weight = "bold"),
        locations = gt::cells_row_groups(groups = everything())
    ) |>
    tab_header(title = md("**Table 3 - Suspected Somatic neurologic diagnoses at follow-up**"))
```

| **Table 3 - Suspected Somatic neurologic diagnoses at follow-up** | |
| --- | --- |
| **Condition** | **N = 708**1 |
| Neuromuscular disease | |
| Any neuromuscular disease | 182 (26%) |
| Polyneuropathy | 126 (18%) |
| Mononeuropathies |  |
| Any mononeuropathy | 62 (8.8%) |
| Peroneal neuropathy | 36 (5.1%) |
| Lateral femoral neuropathy | 13 (1.8%) |
| Other lower limb neuropathies | 5 (0.7%) |
| Median neuropathy | 9 (1.3%) |
| Ulnar neuropathy | 7 (1.0%) |
| Motor neuron disease | 2 (0.3%) |
| Radiculopathy | 5 (0.7%) |
| Cerebrovascular disease | |
| Cerebrovascular disease | 73 (10%) |
| Epilepsy | |
| Epilepsy | 19 (2.7%) |
| Other diagnoses | |
| Parkinsonism | 6 (0.8%) |
| Tremor | 17 (2.4%) |
|  |  |
| --- | --- |
| 1 n (%) | |

# Symptoms modeling

```
# build data ------------------------------------------------------
symptoms_glm_data <-
    bind_cols(
        questions |>
            select(Q4_speech.fup, Q7_weakness.fup, Q8_walk.fup, Q9_paresthesia.fup) |>
            mutate(across(everything(), \(x) x %in% "yes")),   # outcomes: logical
        df |>
            select(sex, age, diabetes, hypertension, stroke, los_total, icu_care, intubation) |>
            mutate(
                across(c(diabetes, hypertension, stroke, icu_care, intubation),
                       \(x) if (is.logical(x)) as.numeric(x)),   # predictors: logical
                sex = forcats::fct_relevel(sex, "male")         # ref = male
            )
    )

symptoms_glm_data <- 
    questions |> 
    select(c(Q4_speech.fup,
             Q7_weakness.fup,
             Q8_walk.fup,
             Q9_paresthesia.fup)) |> 
    bind_cols(df |> 
                  select(sex, 
                         age, 
                         diabetes,
                         hypertension,
                         stroke,
                         los_total,
                         icu_care,
                         intubation,
                  ))

symptoms_glm_data <-
    symptoms_glm_data |>
    mutate(across(matches("Q"), \(x) x %in% "yes")) |> 
    mutate(across(where(is.logical), as.numeric)) |> 
    mutate(sex = as.factor(sex)) |> 
    mutate(sex = relevel(sex, ref = "female"))


# outcomes & predictors ------------------------------------------
symptoms_outcomes   <- names(select(symptoms_glm_data, starts_with("Q")))
symptoms_predictors <- names(select(symptoms_glm_data, -all_of(symptoms_outcomes)))

# fit models ------------------------------------------------------
formulas <- 
    set_names(symptoms_outcomes) |>
    map(\(y) reformulate(symptoms_predictors, response = y))

symptoms_models <- map(formulas, \(f) glm(f,
                                          data = symptoms_glm_data,
                                          family = binomial()))

# pretty names for the outcomes
symptoms_fitted_names <- 
    c("Speech impairment",
      "Muscle Weakness",
      "Gait impairment",
      "Paresthesias")

symptoms_logistic_regression_table <- 
    map(
    symptoms_models,
    \(x) tbl_regression(
        x,
        exponentiate = TRUE,
        show_single_row = everything(),
        label = list(
            age ~ "Age",
            sex ~ "Sex - male",
            diabetes ~ "Diabetes",
            hypertension ~ "Hypertension",
            stroke ~ "Previous stroke",
            los_total ~ "Lenght of stay",
            icu_care ~ "Intensive Care admission",
            intubation ~ "Intubation"
        )
    ) |>
        modify_header(label ~ "**Predictor**") |>
        bold_p(t = 0.05) |>
        modify_column_indent(columns = label, double_indent = TRUE)
) |>
    tbl_merge(tab_spanner = symptoms_fitted_names) |> 
    as_gt() |> 
    tab_header(
        title = md(
            "**Logistic Regression Analysis of Predictors for Neurological Symptoms**")
    )

symptoms_logistic_regression_table
```

| **Logistic Regression Analysis of Predictors for Neurological Symptoms** | | | | | | | | | | | | |
| --- | --- | --- | --- | --- | --- | --- | --- | --- | --- | --- | --- | --- |
| **Predictor** | Speech impairment | | | Muscle Weakness | | | Gait impairment | | | Paresthesias | | |
|  | **OR** | **95% CI** | **p-value** | **OR** | **95% CI** | **p-value** | **OR** | **95% CI** | **p-value** | **OR** | **95% CI** | **p-value** |
| Sex - male | 0.86 | 0.53, 1.40 | 0.5 | 0.94 | 0.61, 1.44 | 0.8 | 1.06 | 0.68, 1.66 | 0.8 | 0.87 | 0.60, 1.25 | 0.4 |
| Age | 1.00 | 0.98, 1.02 | 0.8 | 0.99 | 0.97, 1.00 | 0.2 | 1.00 | 0.98, 1.02 | 0.8 | 0.99 | 0.98, 1.01 | 0.3 |
| Diabetes | 1.43 | 0.84, 2.40 | 0.2 | 1.56 | 0.98, 2.48 | 0.062 | 2.07 | 1.29, 3.33 | 0.003 | 1.55 | 1.04, 2.32 | 0.031 |
| Hypertension | 1.03 | 0.59, 1.84 | >0.9 | 0.93 | 0.56, 1.54 | 0.8 | 1.11 | 0.66, 1.90 | 0.7 | 1.14 | 0.74, 1.77 | 0.5 |
| Previous stroke | 1.77 | 0.61, 4.46 | 0.3 | 1.12 | 0.38, 2.85 | 0.8 | 3.95 | 1.67, 9.09 | 0.001 | 2.21 | 0.98, 4.84 | 0.050 |
| Lenght of stay | 1.02 | 1.01, 1.04 | 0.003 | 1.02 | 1.01, 1.04 | <0.001 | 1.03 | 1.01, 1.04 | <0.001 | 1.02 | 1.00, 1.03 | 0.007 |
| Intensive Care admission | 0.45 | 0.20, 0.93 | 0.039 | 1.50 | 0.80, 2.78 | 0.2 | 1.54 | 0.77, 3.05 | 0.2 | 0.92 | 0.52, 1.59 | 0.8 |
| Intubation | 1.09 | 0.50, 2.51 | 0.8 | 0.93 | 0.52, 1.69 | 0.8 | 1.32 | 0.72, 2.51 | 0.4 | 1.55 | 0.90, 2.71 | 0.12 |
|  |  |  |  |  |  |  |  |  |  |  |  |  |
| --- | --- | --- | --- | --- | --- | --- | --- | --- | --- | --- | --- | --- |
| Abbreviations: CI = Confidence Interval, OR = Odds Ratio | | | | | | | | | | | | |

# Global models

```
# create a list of formulas, one for every outcome
 who_vs_diagnoses_formulas <- 
   map(diagnoses_names,
       \(x) reformulate(response = x,
                        termlabels = predictors_names
                        )
       ) |> 
   set_names(diagnoses_names)


# Refit models with proper Firth logistic regression with logistf package
who_vs_diagnoses_fitted <- map(who_vs_diagnoses_formulas, 
                               \(formula) logistf(formula, who_vs_diagnoses))

# pretty names for the outcomes
symptoms_fitted_names <- 
    c("Speech impairment",
      "Muscle Weakness",
      "Gait impairment",
      "Paresthesias")

# pretty table for publication
table_glm_who_vs_diagnoses <- 
    map(who_vs_diagnoses_fitted, \(x) {
        tbl_regression(x,
                       exponentiate = TRUE,
                       show_single_row = everything(),
                       estimate_fun = \(x) ifelse(x>200,
                                                  ">200",
                                                  style_ratio(x)),
        label = list(
            who_arms_extended ~ "Arms extended", 
            who_match_stick ~ "match stick test",
            who_cloth_gross_sensation ~ "cloth sensation test",
            who_index_nose ~ "index-nose test",
            who_tandem ~ "tandem walk test",
            who_balance ~ "balance",
            who_romberg ~ "Romberg's test",
            Q1_loc.fup ~ "Loss of consciousness",
            Q2_contact.fup ~ "Loss of contact",
            Q3_tremors_spasms.fup ~ "Tremors/spasms",
            Q4_speech.fup ~ "Speech impairment",
            Q5_face_pain.fup ~ "Facial pain",
            Q6_face_paral.fup ~ "Facial paralysis",
            Q7_weakness.fup ~ "Muscle weakness",
            Q8_walk.fup ~ "Gait impairment",
            Q9_paresthesia.fup ~ "Paresthesias",
            Q10_headache.fup ~ "Headache")) |>
    bold_p(t = 0.05) |>
    modify_column_indent(columns = label, double_indent = TRUE) |>
  modify_header(estimate ~ "**OR**") 
})

table_glm_who_vs_diagnoses |> 
    tbl_merge(tab_spanner = str_replace_all(diagnoses_names, "_", " ")) |> 
    as_gt() |> 
    tab_row_group(
        group = "Triage physical exam",
        rows = 1:7) |> 
    tab_row_group(
        group = "Questionnaire",
        rows = 8:17) |> 
  tab_header(
    title = md("**Logistic Regression Analysis of WHO Protocol Items for Suspected Neuromuscular Disease, Epilepsy, and Cerebrovascular Disease**"))
```

| **Logistic Regression Analysis of WHO Protocol Items for Suspected Neuromuscular Disease, Epilepsy, and Cerebrovascular Disease** | | | | | | | | | |
| --- | --- | --- | --- | --- | --- | --- | --- | --- | --- |
| **Characteristic** | Neuromuscular disease | | | Epilepsy | | | Cerebrovascular disease | | |
|  | **OR** | **95% CI** | **p-value** | **OR** | **95% CI** | **p-value** | **OR** | **95% CI** | **p-value** |
| Questionnaire | | | | | | | | | |
| Loss of consciousness | 0.82 | 0.24, 2.47 | 0.7 | 3.56 | 0.55, 17.9 | 0.2 | 2.83 | 0.74, 9.19 | 0.12 |
| Loss of contact | 0.93 | 0.32, 2.58 | 0.9 | 16.5 | 4.02, 63.0 | <0.001 | 1.02 | 0.23, 3.30 | >0.9 |
| Tremors/spasms | 1.04 | 0.53, 1.98 | >0.9 | 0.97 | 0.18, 4.59 | >0.9 | 0.68 | 0.25, 1.64 | 0.4 |
| Speech impairment | 0.94 | 0.48, 1.76 | 0.8 | 0.61 | 0.06, 3.36 | 0.6 | 0.90 | 0.38, 1.95 | 0.8 |
| Facial pain | 0.90 | 0.29, 2.67 | 0.9 | 0.64 | 0.00, 7.98 | 0.8 | 0.73 | 0.13, 2.82 | 0.7 |
| Facial paralysis | 0.38 | 0.05, 2.32 | 0.3 | 0.06 | 0.00, 4.62 | 0.2 | 10.2 | 1.53, 72.2 | 0.018 |
| Muscle weakness | 1.83 | 1.04, 3.20 | 0.036 | 1.03 | 0.19, 4.07 | >0.9 | 1.12 | 0.51, 2.33 | 0.8 |
| Gait impairment | 1.32 | 0.73, 2.37 | 0.4 | 1.38 | 0.33, 5.23 | 0.6 | 1.45 | 0.67, 3.05 | 0.3 |
| Paresthesias | 6.45 | 4.23, 9.91 | <0.001 | 0.13 | 0.01, 0.73 | 0.018 | 1.39 | 0.74, 2.54 | 0.3 |
| Headache | 0.80 | 0.53, 1.20 | 0.3 | 1.68 | 0.57, 4.92 | 0.3 | 0.84 | 0.48, 1.45 | 0.5 |
| Triage physical exam | | | | | | | | | |
| Arms extended | 1.00 | 0.32, 3.00 | >0.9 | 2.88 | 0.22, 27.6 | 0.4 | 1.45 | 0.42, 4.56 | 0.5 |
| match stick test | 0.90 | 0.36, 2.22 | 0.8 | 0.80 | 0.03, 8.45 | 0.9 | 1.87 | 0.70, 4.69 | 0.2 |
| cloth sensation test | 0.62 | 0.21, 1.72 | 0.4 | 2.79 | 0.29, 17.4 | 0.3 | 1.20 | 0.37, 3.40 | 0.7 |
| index-nose test | 0.88 | 0.32, 2.28 | 0.8 | 1.12 | 0.13, 7.28 | >0.9 | 2.38 | 0.83, 6.26 | 0.10 |
| tandem walk test | 3.27 | 1.92, 5.60 | <0.001 | 2.28 | 0.52, 8.49 | 0.3 | 1.74 | 0.85, 3.46 | 0.13 |
| balance | 0.39 | 0.09, 1.61 | 0.2 | 1.98 | 0.13, >200 | 0.7 | 1.33 | 0.33, 6.37 | 0.7 |
| Romberg's test | 1.74 | 0.51, 5.88 | 0.4 | 1.24 | 0.01, 12.3 | 0.9 | 1.11 | 0.25, 3.84 | 0.9 |
|  |  |  |  |  |  |  |  |  |  |
| --- | --- | --- | --- | --- | --- | --- | --- | --- | --- |
| Abbreviation: CI = Confidence Interval | | | | | | | | | |

# Best subsets - logistic regression with Firth correction

\*\* Warning - This can take a long time to compute. Consider caching results \*\*

```
use_cached_results_from_best_subsets <- FALSE
```

## Options for iterative testing

```
# define pool of predictors to evaluate
# predictors_names <- predictors_names[1:10]

# If set to use cached results from best subsets previous run, skip
if (use_cached_results_from_best_subsets) {
  cat(md("**Skipping heavy computations for best subsets. Using cached results**"))
print(rio::import(here("intermediary_data", "best_subsets_final_table.rds")))
  knitr::knit_exit()
}

# define whether to write results to disk
write_to_disk <- TRUE
```

## Definition of parallel model fitting function

```
best_subsets_grid_selection <- function(dataset,
                                        num_workers = parallel::detectCores() - 1,
                                        outcome,
                                        predictors,
                                        by = "BIC",
                                        use_firth = FALSE,
                                        future_plan = c("sequential",
                                                        "multisession",
                                                        "multicore",
                                                        "mclapply"),
                                        formula_storage_class = "character"
) {
    
    library(parallel)
    library(furrr)
    library(purrr)
    library(tidyr)
    library(dplyr)
    if (use_firth) library(brglm2)
    
    # Ensure future_plan is one of the four options
    future_plan <- match.arg(future_plan)
    
    # Ensure formula_storage_class is one of the two options
    formula_storage_class <- match.arg(formula_storage_class,
                                       choices = c("character", "formula"))
    
    # Check arguments
    if (!is.data.frame(dataset)) {
        stop("dataset must be a data frame or coercible to a data.frame")
    }
    if (!is.character(outcome) | !is.character(predictors)) {
        stop("outcome and predictors must be character")
    }
    if (!outcome %in% colnames(dataset)) {
        stop("outcome must be the name of a potential outcome variable")
    }
    if (!all(predictors %in% colnames(dataset))) {
        stop("predictors must be a character vector with names of predictor variables")
    }
    if (!by %in% c("AIC", "BIC")) {
        stop("by must be Akaike information criterion (AIC) or Bayes information criterion (BIC)")
    }
    
    # Set up the appropriate plan for parallelization
    if (future_plan == "multisession") {
        plan(multisession, workers = num_workers)
    } else if (future_plan == "multicore") {
        plan(multicore, workers = num_workers)
    }
    
    # Generate combinations of predictors
    predictors_combinations <- map(seq_along(predictors),
                                   \(x) combn(predictors,
                                              x,
                                              simplify = FALSE)
                                   ) |>
        flatten()
    
    # Create formulas for each combination
    formulas <- map(predictors_combinations,
                    \(x) reformulate(response = outcome, termlabels = x))
    
    # Function to fit the GLM and capture warnings
    model_fit <- function(formula, dataset, storage_class) {  
        warning_msg <- NULL
        
        fit <- tryCatch(
            {
                if (use_firth) {
                    # Firth logistic regression using brglm2
                    glm(
                        formula = formula,
                        data = dataset,
                        family = "binomial",
                        method = "brglmFit",
                        type = "AS_mean"
                    )                } else {
                    # Regular GLM
                    glm(formula = formula,
                        data = dataset,
                        family = "binomial")
                }
            },
            warning = function(w) {
                warning_msg <<- w$message
                suppressWarnings(glm(formula = formula,
                                     data = dataset,
                                     family = "binomial"))
            },
            error = function(e) {
                return(NULL)
            }
        )
        
        if (!is.null(fit)) {
            if (storage_class == "character") {
                # Convert formula to single character string
                formula_stored <- paste(deparse(formula), collapse = " ")
            } else {
                # Keep formula as a formula object
                formula_stored <- formula
            }
            
            output <- tibble(
                formula = formula_stored,
                AIC = AIC(fit),
                BIC = BIC(fit),
                warnings = warning_msg
            )
        } else {
            if (storage_class == "character") {
                # Convert formula to single character string
                formula_stored <- paste(deparse(formula), collapse = " ")
            } else {
                # Keep formula as a formula object
                formula_stored <- formula
            }
            
            output <- tibble(
                formula = formula_stored, 
                AIC = NA,
                BIC = NA,
                warnings = "Error in model fit"
            )
        }
        
        # Output
        return(output)
    }
    
    # Define how to handle different future plans
    if (future_plan == "sequential") {
        # Use vanilla map (sequential execution)
        list_of_models <- map(formulas,
                              \(x) model_fit(x, dataset, formula_storage_class)) 
    } else if (future_plan == "mclapply") {
        # Use mclapply for parallel execution (Unix systems only)
        list_of_models <- mclapply(formulas,
                                   \(x) model_fit(x, dataset, formula_storage_class),
                                   mc.cores = num_workers)
    } else {
        # Use future_map for multisession/multicore parallelization
        list_of_models <- future_map(formulas,
                                     \(x) model_fit(x, dataset, formula_storage_class),
                                     .options = furrr_options(seed = TRUE))
    }
    
    # Combine results and arrange by the chosen criterion (AIC or BIC)
    list_of_models <- bind_rows(list_of_models)
    
    # Arrange by the chosen criterion (AIC or BIC)
    list_of_models |> arrange(!!sym(by))
}
```

# Extensive grid fitting of all potential models

```
# set plan for the three calls to best_glms_grid_search()
# Detect OS and assign parallel plan (mclapply for unix, multisession for windows)
if (.Platform$OS.type == "unix") {
    parallel_plan <- "mclapply"  # For Unix-like systems (Linux/macOS)
} else if (.Platform$OS.type == "windows") {
    parallel_plan <- "multisession"  # For Windows
}

# determine number of working cpu threads (save 2 threads for system)
num_threads <- parallel::detectCores() -2

# create a list of models for every diagnosis

# epilepsy
best_subsets_epilepsy <- 
    best_subsets_grid_selection(dataset = who_vs_diagnoses,
                                num_workers = num_threads,
                                outcome = "Epilepsy",
                                future_plan = parallel_plan,
                                predictors = predictors_names,
                                by = "BIC",
                                use_firth = TRUE,
                                formula_storage_class = "character")

# neuromuscular
best_subsets_neuromuscular <- 
    best_subsets_grid_selection(dataset = who_vs_diagnoses,
                                num_workers = num_threads,
                                outcome = "Neuromuscular_disease",
                                future_plan = parallel_plan,
                                predictors = predictors_names,
                                use_firth = FALSE,
                                by = "BIC",
                                formula_storage_class = "character")

# cerebrovascular
best_subsets_cerebrovascular <- 
    best_subsets_grid_selection(dataset = who_vs_diagnoses,
                                num_workers = num_threads,
                                outcome = "Cerebrovascular_disease",
                                future_plan = parallel_plan,
                                predictors = predictors_names,
                                use_firth = TRUE,
                                by = "BIC",
                                formula_storage_class = "character")

# write all files to disk as parquet files

if(write_to_disk){
export(best_subsets_epilepsy,
       file = here("intermediary_data", "best_subsets_epilepsy_firth.csv"))

export(best_subsets_neuromuscular,
       file = here("intermediary_data", "best_subsets_neuromuscular_firth.csv"))

export(best_subsets_cerebrovascular,
       file = here("intermediary_data", "best_subsets_cerebrovascular_firth.csv"))
}
```

# Reporting final models

```
rename_labels_best_subsets <- c(
    who_arms_extended = "Arms extended",
    who_match_stick = "Match stick test",
    who_cloth_gross_sensation = "Cloth sensation test",
    who_index_nose = "Index–nose test",
    who_tandem = "Tandem walk test",
    who_balance = "Balance",
    who_romberg = "Romberg's test",
    Q1_loc.fup = "Loss of consciousness",
    Q2_contact.fup = "Loss of contact",
    Q3_tremors_spasms.fup = "Tremors/spasms",
    Q4_speech.fup = "Speech impairment",
    Q5_face_pain.fup = "Facial pain",
    Q6_face_paral.fup = "Facial paralysis",
    Q7_weakness.fup = "Muscle weakness",
    Q8_walk.fup = "Gait impairment",
    Q9_paresthesia.fup = "Paresthesias",
    Q10_headache.fup = "Headache"
)

# create list of outcomes with lists of all model fit metrics and formulas
best_subsets <- 
    list(
        cerebrovascular = best_subsets_cerebrovascular,
        neuromuscular = best_subsets_neuromuscular,
        epilepsy = best_subsets_epilepsy)

# select top model for each outcome
single_best_model_formulas <- 
    best_subsets |> 
    map(\(x) slice_head(x, n = 1)) |> 
    map(\(x) pull(x, formula))

models <- imap(single_best_model_formulas, \(formula, name) {
  logistf(
    data = who_vs_diagnoses,
    formula = as.formula(formula)
  )
})

tables <- map(models, \(x) {
  vars <- attr(terms(x), "term.labels")

  # subset to existing predictors
  valid_labels <- rename_labels_best_subsets[names(rename_labels_best_subsets) %in% vars]

  # convert to a named list
  label_list <- as.list(valid_labels)

  tbl_regression(
    x,
    exponentiate = TRUE,
    show_single_row = everything(),
    label = label_list
  ) |>
    modify_header(estimate ~ "**OR**") |>
    bold_p(t = 0.05) |>
    modify_column_indent(columns = label, double_indent = TRUE)
})

# Merge tables with appropriate spanner labels
best_subsets_final_table <- tbl_merge(
    tbls = tables,
    tab_spanner = c(
        "Cerebrovascular disease",
        "Neuromuscular disease",
        "Epilepsy" 
    )
) |> 
    as_gt() |> 
    tab_header(
        title = md("**Table - Logistic regression analysis: major diagnoses and who protocol**"),
        subtitle = md("**Simplified Models From Best Subsets Selection**"))

# print final table
best_subsets_final_table
```

| **Table - Logistic regression analysis: major diagnoses and who protocol** | | | | | | | | | |
| --- | --- | --- | --- | --- | --- | --- | --- | --- | --- |
| **Simplified Models From Best Subsets Selection** | | | | | | | | | |
| **Characteristic** | Cerebrovascular disease | | | Neuromuscular disease | | | Epilepsy | | |
|  | **OR** | **95% CI** | **p-value** | **OR** | **95% CI** | **p-value** | **OR** | **95% CI** | **p-value** |
| Index–nose test | 3.49 | 1.56, 7.55 | 0.003 |  |  |  |  |  |  |
| Tandem walk test | 2.94 | 1.68, 5.08 | <0.001 | 3.38 | 2.20, 5.20 | <0.001 | 4.11 | 1.49, 11.1 | 0.007 |
| Facial paralysis | 12.8 | 2.40, 82.5 | 0.003 |  |  |  |  |  |  |
| Paresthesias |  |  |  | 7.50 | 5.02, 11.3 | <0.001 | 0.09 | 0.01, 0.45 | 0.001 |
| Loss of contact |  |  |  |  |  |  | 27.0 | 8.42, 86.9 | <0.001 |
|  |  |  |  |  |  |  |  |  |  |
| --- | --- | --- | --- | --- | --- | --- | --- | --- | --- |
| Abbreviation: CI = Confidence Interval | | | | | | | | | |

```
if(write_to_disk){
export(best_subsets_final_table,
       file = here("intermediary_data", "best_subsets_final_table.rds"))
}
```

# Exploratory analysis - epilepsy vs tandem walk

```
data_for_fishers <- bind_cols(tandem = who_physical$`Tandem walk test`,
                              coordination = df$coordination
                              )
data_for_fishers |> mutate(coordination = str_detect(coordination, "ataxia"),
                           tandem = !tandem %in% "normal") |> 
    table() |> 
    stats::fisher.test()
```

```
    Fisher's Exact Test for Count Data

data:  table(mutate(data_for_fishers, coordination = str_detect(coordination, "ataxia"), tandem = !tandem %in% "normal"))
p-value = 9.849e-12
alternative hypothesis: true odds ratio is not equal to 1
95 percent confidence interval:
  5.251869 26.257042
sample estimates:
odds ratio 
  11.36535
```
