# Supplementary material for: Neurological symptoms and physical exam findings 6–11 months post-COVID-19: a cohort study
Source: Sci Rep. 2026 Jan 2;16:3732. doi: 10.1038/s41598-025-33779-w (PMC12852936; doi:10.1038/s41598-025-33779-w)
Supplement: Supplementary file 4 — Supplementary Material 4 [file 41598_2025_33779_MOESM4_ESM.pdf]

Supplementary Figure 1. Study Flowchart

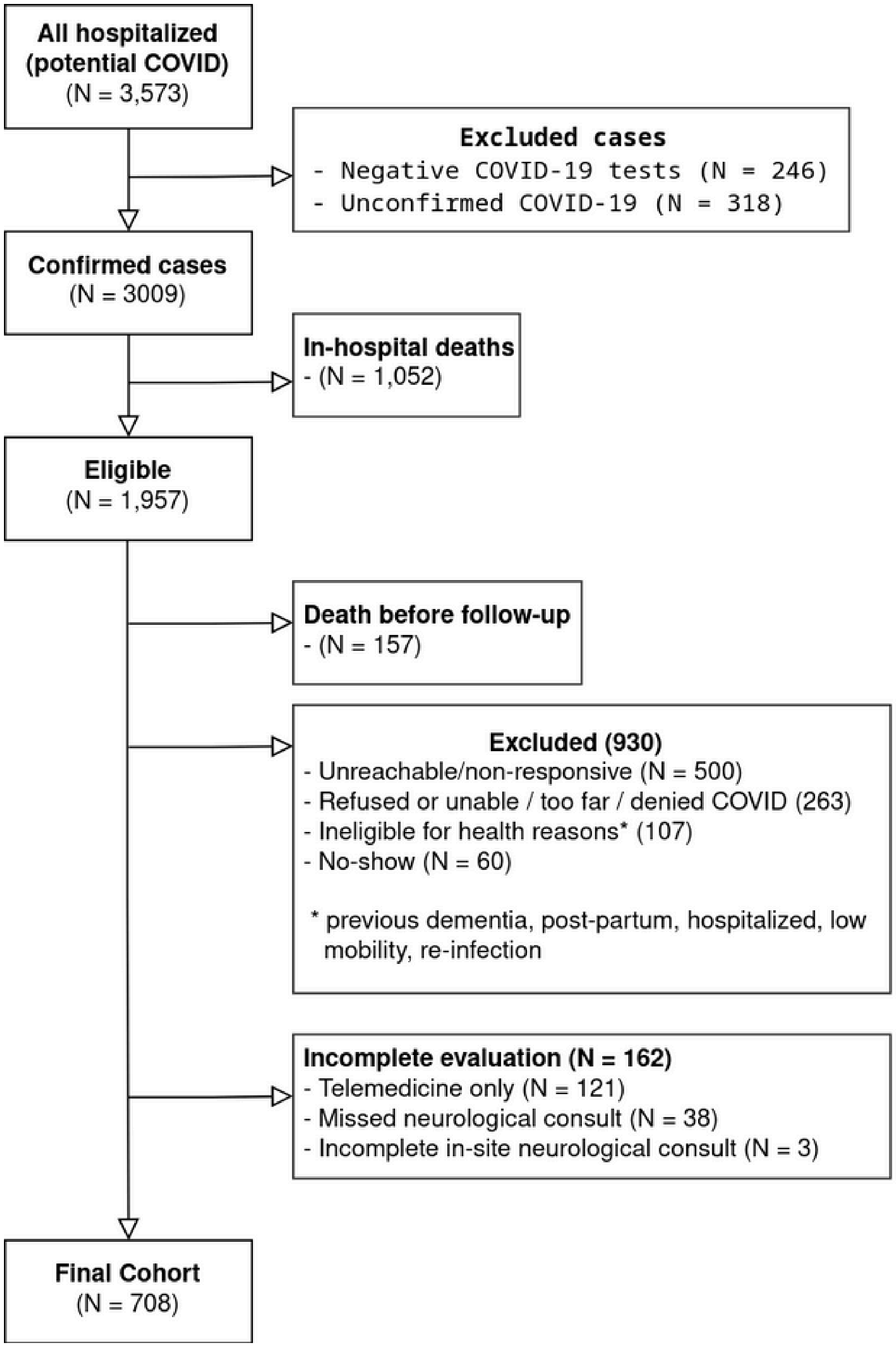

Figure Caption: Flow diagram of participant inclusion, exclusion, and follow-up for the post-COVID neurological cohort, resulting in a final analytic sample of 708 patients.

Supplementary Figure 2. Association Matrix (Cramér's V) - WHO Protocol

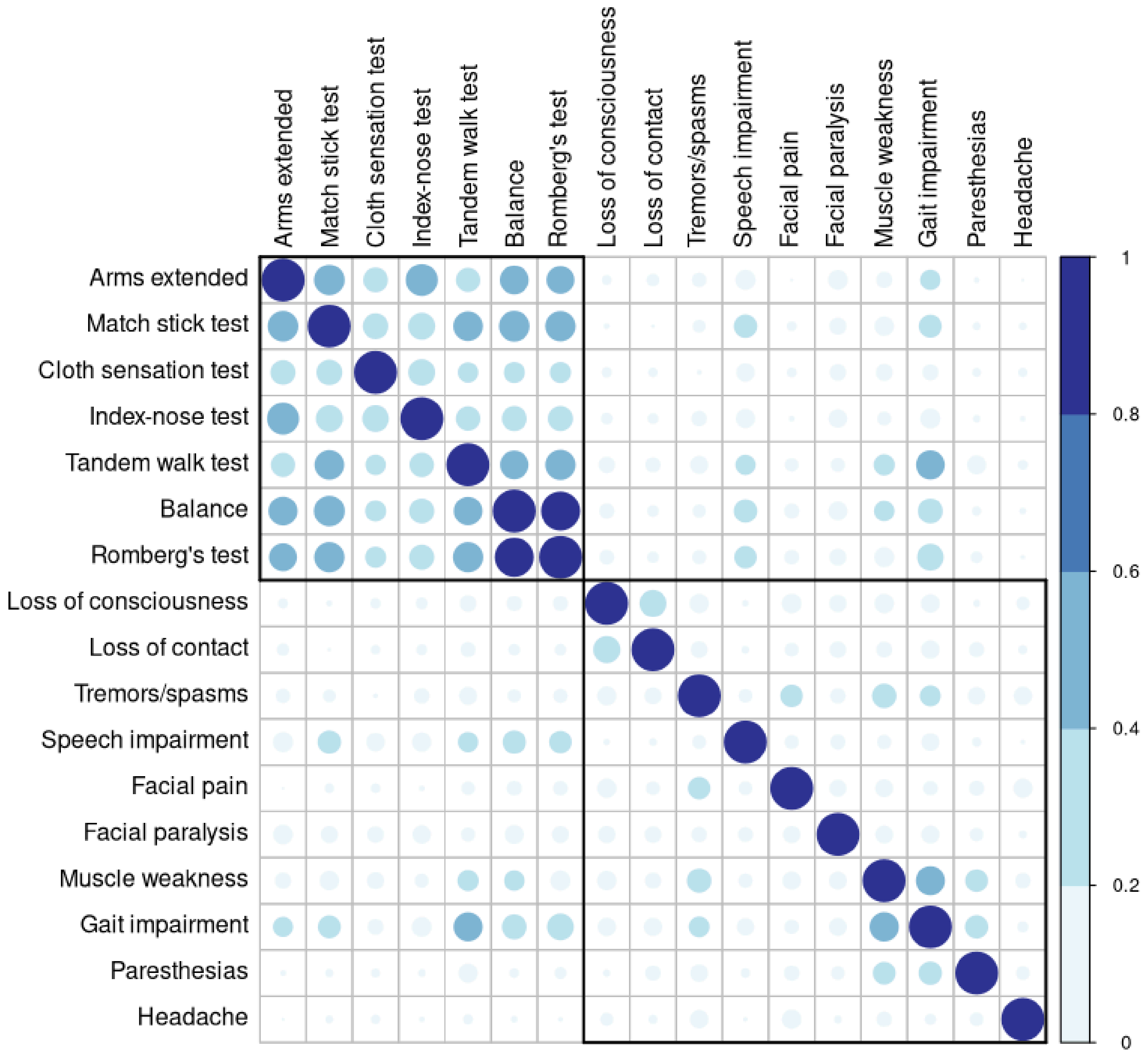

Figure Caption: Matrix of pairwise Cramér’s V for all 17 items of the WHO protocol. Rectangles group the questionnaire and physical examination steps
